# Effects of sulfur on the growth and metabolism of sulfur and nitrogen in cabbage

**DOI:** 10.1371/journal.pone.0359848

**Published:** 2026-11-11

**Authors:** YuMei Zhao, Yu Yang, YunDan Deng, XiaoHong Chai, LuShan Li

**Affiliations:** 1 Faculty of Landscape Architecture and Horticulture, Southwest Forestry University, Kunming, Yunnan, China; 2 Yunnan Key Laboratory of Landscape Plant Resource Cultivation and Application, Kunming, Yunnan, China; 3 Cooperative College, Jiangsu Vocational College of Business, Nantong, Jiangsu, China; Center for Research and Technology Transfer, VIET NAM

## Abstract

Sulfur the fourth essential nutrient element required for plants after nitrogen, phosphorus and potassium plays a crucial physiological role in plant growth and stress resistance. This study aims to investigate the influence of sulfur on cabbage growth. In this study, cabbage was treated with sulfur-free Hoagland nutrient solution (control; − S; 0 mmol//L),or normal Hoagland nutrient solution(treatment; + S; 2 mmol/L). By measuring the cabbage growth index, antioxidant system, Nitrogen and Sulffur metabolism at different periods, this study explored S’s regulation mechanism for growth, nitrogen metabolism and sulfur metabolism. The results showed that sulfur could significantly promote the growth of cabbage. Under + S treatment, plant height and stem diameter were 15.1% and 15.6% higher than that of -S treatment, and the root dry matter distribution rate was the highest at the rosette and heading stage. 15.47% and 13.09% under + S treatment; 10.15% and 10.4% under -S treatment. Additionally, sulfur promoted photosynthesis, fluorescence, antioxidant enzyme system and glutathione cycle of cabbage. During the period when the Ci was the highest and the Pn reached its maximum, the Ci of + S treatment was 15% higher than that of -S treatment. We discovered that sulfur promoted both nitrogen and sulfur metabolism in cabbage, and + S treatment significantly increased the activities of ATP sulfase (ATPS) and APS reductase (APR) in cabbage. During the whole growth period of cabbage, + S treatment increased ATPS activity by 15.5%, 13.8%, 45.2% and 25.7%, respectively. To sum up, sulfur not only participates in the regulation of basal metabolism during cabbage growth and development but also plays a imperative role in key physiological processes such as activation of the antioxidant system, optimization of the photosynthetic efficiency, and synergistic metabolism of nitrogen and sulfur assimilation. This study provides a theoretical basis for a indepth understanding of the molecular mechanisms by which sulfur nutrition regulates crop growth and metabolic networks, and also lays an important foundation for the precision application of sulfur fertilizers and the optimization of crop sulfur nutrition management strategies.

## Introduction

Sulfur is the fourth essential nutrient element for plants after nitrogen, phosphorus and potassium, and its absorption in plants is second only to that of nitrogen [1]. The sulfur absorption of cruciferous crops, especially Brassica crops, is higher than that of some grasses and more than three times the number of family crops. Sulfur exists in various forms and is abundant in the environment. It can be absorbed and utilized by plants, and plays a critical role in their growth and development. Research has demonstrated that sulfur plays essential physiological roles in plant growth, development, and stress resistance mechanisms [2]. Its metabolic regulation spans the entire plant life cycle, from seed germination to reproductive growth [3]. During the seed germination stage, sulfur participates in post-translational modifications of proteins by synthesizing sulfur-containing amino acids (such as cysteine and methionine), particularly by forming disulfide bonds to stabilize the spatial conformation of proteolytic enzymes, thereby enhancing protease activity, promoting the breakdown of storage proteins in seeds. This provides essential amino acids and nitrogen sources for rapid embryonic growth [4]. During the vegetative growth stage, sulfur is a key component of the iron-sulfur (Fe-S) cluster proteins and the thioredoxin system. The former plays a central role in the electron transport chain of Photosystem I (PSI), while the latter indirectly drives chlorophyll biosynthesis by regulating the redox status of chloroplastic enzymes [5]. Once sulfur becomes deficient, chlorophyll synthesis is impaired, plants exhibit leaf chlorosis, and photosynthetic efficiency significantly decreases, which in turn limits plant biomass accumulation. During the reproductive growth stage, sulfur is transported over long distances from rosette leaves to primary stems and floral organs via sulfate transporters (such as SULTR2;1). In anthers and pollen, sulfur accumulates abundantly in glutathione (GSH) to maintain the reductive potential required for pollen germination and acts as a signaling molecule to regulate the timing of flowering transition (bolting) [6]. During the late fruit development stage, sulfur serves as a precursor for sulfur-containing secondary metabolites. For example, in Brassicaceae plants, sulfur is incorporated into the structural backbone of glucosinolates, whose degradation products (isothiocyanates) not only impart unique flavors to vegetables but also directly determine fruit quality characteristics [7]. Sulfur serves as a critical structural component of key plant enzymes and proteins, as well as a core element for chlorophyll functionality [8]. Its physiological roles are multifaceted, directly promoting plant protein synthesis and enhancing photosynthetic efficiency, thereby increasing crop yield and improving nutritional quality [9–11]. Extensive studies have investigated sulfur’s effects on staple crops such as maize [12], wheat [13], soybeans [14], and peanuts [15], with broader implications for agricultural productivity [16].

Recent research has expanded to vegetable crops, revealing synergistic interactions between sulfur and nitrogen assimilation pathways. These elements exhibit interdependent metabolic regulation, jointly influencing protein synthesis and enzyme activity [17]. In cruciferous crops, sulfur limitation disrupts the production of proteins and secondary metabolites, while optimal sulfur supplementation enhances nitrogen utilization and yield, as also demonstrated in wheat systems [18]. Studies in Chinese cabbage (Brassica rapa) have identified 19 BrSULTR members that are expressed in respond to salt stress and low-temperature stress [19]. Hooi et al. found that an anti-ESP antibody reacted with both the 39 and 35 kDa ESP forms in Brassica napus and strongly with a polypeptide corresponding to the 35 kDa ESP form in Crambe abyssinica [20]. Ma Xiaoli et al. discovered that H_2_S selectively S-sulfhydrates the flowering repressor BraFLCs in Chinese cabbage [21]. Despite these advances, significant knowledge gaps persist regarding sulfur’s influence on leafy vegetables, particularly in understanding growth dynamics and dry matter accumulation, the regulatory mechanisms of the glutathione cycle, sulfur-nitrogen metabolic interactions, and stress adaptation responses. Underscoring warrants further exploration to fully elucidate sulfur’s role in leafy vegetable physiology and productivity.

In this study, ‘Zhonggan 11’ was used as the experimental material. By observing and measuring the effects of sulfur-sufficient (+S) and sulfur-deficient (-S) conditions on plant growth parameters, dry matter allocation, photosynthetic chlorophyll fluorescence, antioxidant enzyme system, glutathione cycle dynamics, nitrogen and sulfur assimilation enzymes, and key regulatory genes in the sulfur assimilation pathway, this study provides fundamental insights into sulfur-mediated regulation of crop growth and establishes a theoretical framework for research on the glucosinolate biosynthesis pathway in Brassicaceae crops.

## Materials and methods

### Plant and experimental design

This experiment was carried out in a modern greenhouse (Daytime: 25–30 °C; Nighttime: 16–20 °C) at Gansu Agricultural University (coordinates: N36°05′39.86″, E103°42′31.09″), with ‘Zhonggan 11’ as the test cultivar. The seeds were stirred in warm water at 55 ℃ for 15 min, then sowed in plug trays, and transplanted into nutrient pots (vermiculite as substrate:pearlite = 2:1). The treatment was started after 6 days of slow seedling, and the nutrient solution was irrigated once every 10 days. The concentration of 2 mmol·L ⁻ ¹ corresponds to the standard sulfur supply in the widely used Hoagland nutrient solution formulation. This concentration has been consistently employed in our preliminary cultivation practices and is recognized as a sufficient and non-toxic sulfur level that supports normal growth for a broad range of plant species. Therefore, we selected this concentration as the “sufficient” treatment in our experimental design. The control group (CK) was treated with modified sulfur-free Hoagland nutrient solution (Ca(NO _3_)_2_ 1.75 mmol L^-1^, KNO_3_ 3 mmol L^-1^, NH_4_H_2_PO_4_ 0.5 mmol L^-1^, MgCl_2_•7H _2_O 2 mmol L^-1^), and the T1 group (T1) was treated with Hoagland nutrient solution (Ca(NO_3_)_2_ 1.75 mmol L^-1^, KNO_3_ 3 mmol L^-1^, NH _4_H _2_PO _4_ 0.5 mmol L −1, MgSO_4_•7H _2_O 2 mmol L ^−1^). The irrigation condition was applied based on soil nutrient condition, and the water was poured thoroughly at one time. The growth index, antioxidant enzymes, photosynthetic parameters, gas exchange parameters, glutathione cycle system, and sulfur metabolism and nitrogen metabolism enzyme system of cabbage were measured at the seedling, rosette, heading and mature stage. The third true leaf of the cabbage was sampled. All cabbage plants were arranged in a randomized block design with three replicates.

### Growth index determination

Plant height measurement: The length of the cord was measured with a ruler from the base of the cabbage stem to the top growing point.

Stem diameter measurement: Stem diameter was measured at the base of cabbage stem using vernier caliper.

Plant morphology: The treated cabbage was placed on a black background board and photographed with a digital camera mounted on a tripod.

### Antioxidant enzyme assay

Extraction of crude enzyme solution: 0.1 g of cabbage leaves was weighed for each treatment, placed in a pre-cooled mortar. Then, 1 mL of extraction solution was added into the mortar and the mixture was homogenized in ice bath. The homogenate was centrifuged at 8,000 rpm at 4 ℃ for 10 min, and the supernatant was collected for enzyme activity determination after centrifugation [22].

Peroxidase (POD): The working solution was prepared according to the instructions of the kit, the wavelength of the microplate reader was adjusted to 470 nm, and the instrument was zeroed using the distilled water. Absorbance values were recorded at 1 min and 2 min, and POD activity was calculated as POD (U.g^-1^)=(A2 − A1)× Vtotal ÷ (W× Vsample ÷ Vsample total) ÷0.005 ÷ T = 4,000 × ∆A ÷ W.

Catalase (CAT): The working solution was prepared according to the instructions of the kit. Corresponding reagents was added to the control tube and the determination tube. the wavelength of the microplate reader was adjusted to 405 nm, and determine the absorbance val-ue.Calculate CAT (μmol.min^-1^.g^-1^)=(∆A − 0.0013)÷0.1 × Vinverse total ÷ (W× Vsample ÷ Vsample total) ÷T = 8.9× (∆A − 0.0013) ÷W.

Superoxide dismutase (SOD): Prepare the working solution according to the instructions of the kit, add the corresponding reagents into the control tube and determination tube, mix well, stand at room temperature for 30 min, and determine the absorbance value at 450 nm.Calculate SOD (U.g^-1^)=(inhibition percentage ÷ (1 − inhibition percentage) × Vtotal)÷ (500 × Vsample ÷ Vtotal)=0.04 × inhibition percentage ÷ (1 − inhibition percentage). Percent inhibition =(A control tube −A test tube) ÷A control tube 100%.

Ascorbate peroxidase (APX):Prepare the working solution according to the instructions of the kit, adjust the wavelength of the microplate reader to 290 nm, and measure its absorbance at 10 s and 130 s respectively. Calculate APX (nmol.min^-1^.g^-1^)=∆A÷ (ε × d) × Vtotal ×109÷ (W× Vsample ÷ Vtotal) ÷T = 1786 × ∆A ÷ W.

### Photosynthetic pigment content

In that seedling stage, rosette stage, head stage and mature stage of cabbage, 0.1 g fresh leaves were weighed and put into 10 mL 80% acetone solution to extract photosynthetic pigments.After soaking for 48 hours, the absorbance was measured at 440 nm, 645 nm and 663 nm.The concentration of chlorophyll a (Chla), chlorophyll b (Chlb), chlorophyll and carotenoid in each gram of leaves were calculated by the formula.The chlorophyll content and carotenoid content of cabbage were compared by observing the images of cabbage leaves in seedling stage put into BLT Plant View 100 plant in vivo imaging system [23].

### Gas exchange parameter

During the seedling, rosette, heading and mature stage of cabbage, between 9:00–11:00 AM on a sunny day, three plants with basically the same growth vigor were randomly selected from each treatment, and the plants were cultured with CIRAS-2 (PP System Inc.,Amesbury, MA01913, USA) portable photosynthesis apparatus. Measuring indicators: Net photosynthetic rate, stomatal conductance, intercellular CO2 concentration and transpiration rate. The relative humidity was 75%, the photon flux of leaf chamber was 300 μmol•m-2•s^-1^, and the temperature was 25 ℃ [24].

### Determination of oxidized glutathione and reduced glutathione

The reduced glutathione (GSH) content in tissues was determined using the GSH assay kit  .Weigh 0.1 g of cabbage leaves, add reagent I and grind in an ice bath. Transfer the extract to a 2 mL centrifuge tube after grinding. Keep the liquid level of all test tubes consistent. Centrifuge at 4 ℃ for 10 min at 8,000 rpm. After centrifugation, gently take out the centrifuge tube to avoid shaking. Take the supernatant and place it at 4 ℃ for testing. Determine the GSH content according to the steps specified in the kit. Repeat each group for 3 times.Calculation of GSH content: GSH (μmol•g^-1^ FW)=y× Vsample ÷(Vsample ÷ Vsample total ×W) The content of GSSG in tissues was determined by GSSG assay kit.Weigh 0.1 g of cabbage leaves, add reagent I and grind in ice bath. After grinding, transfer the extract to a 2 mL centrifuge tube. Keep the liquid level of all test tubes consistent. Centrifuge at 4 ℃ for 10 min at 8,000 rpm. After centrifugation, gently take out the centrifuge tube to avoid shaking. Take the supernatant and place it at 4 ℃ for testing.Calculation of GSSG content: GSSG (nmol.g^-1^ FW)=y× Vsample ÷(Vsample ÷ Vsample total ×W) [25].

### Assay of sulfur metabolizing enzymes

ATP sulfurylase (ATPS): Add the sample according to the extraction and determination methods specifired in the kit, determine the absorbance at 450 nm, and calculate the enzyme activity according to the standard curve equation. The methods of Serine acetyltransferase (SAT), O-acetylserine thiol lyase (OASTL), Sulfite reductase (SiR) and APS reductase (APR) were the same as above [26].

### Determination of nitrogen metabolism enzymes

Nitrate reductase (NR): Add the sample according to the extraction and determination methods specified in the kit, determine the absorbance at 450 nm, and calculate the enzyme activity according to the standard curve equation. The methods of Nitrite reductase (NiR), Glutamate synthase (GOGAT) and Glutamine synthetase (GS) were the same as above [27].

### RNA extraction and real-time qRT-PCR

RNA was extracted from the samples using Plant Total RNA Extraction Kit (Tiangen, China).Complementary DNA was synthesized using 2 µL of RNA as template using Fast King cDNA Dispelling RT Super Mixs kit (Tiangen, China) for 17.The qRT-PCR assay was performed using a Light Cycler®480II real-time PCR instrument.SYBR Green Kit (Tiangen, China) for fluorescence quantification in a 20 µL reaction volume containing 2 µL cDNA solution, 10 µL 2*Super Real Pre Mix Plus, 0.6 µL forward and reverse primers, 0.4 µL 50*ROX Reference DyeΔ and 6.4 µL distilled deionized water.Amplification program conditions were: 95 ℃, 15 min, 40 cycles, 95 ℃, 10 s, 60 cycles, 30 s. In qRT-PCR analysis, all primers were designed using Primer Premier 5.0 software based on the cDNA sequences deposited in GenBank. Each primer pair was validated through gradient PCR to confirm the absence of non-specific amplification, as verified by a single peak in the dissociation curve analysis. The amplification efficiency (E) for each primer pair was calculated from the slope of a standard curve generated by a 10-fold serial dilution of pooled cDNA, and all primers used in this study exhibited amplification efficiencies ranging from 92% to 105% (R² > 0.99). All qRT-PCR reactions were performed in triplicate technical repeats, and three independent biological replicates were conducted for each treatment. The relative expression levels of target genes were calculated using the 2^-∆∆CT^ method after normalization to the selected reference gene [28].

**Table:** 

Gene name	Forward Primer (5’ → 3’)	Reverse Primer (5’ → 3’)
APR	TTGGTTGTGCTGTACGCTCCTTG	CCCTTTCACCGCCAGTTTCTCAG
ATPS	TCGGAGGAGACTTCTTGAGATGGG	CACCTTCTCGTGCTGCTTCATCC
SIR	GCTGTGACTGTGCCTACGGATAAC	CCCATACCGCCACAACATAAAGG
SAT	CGGCGGTGGAGGAGGATGAG	GGGGAGTAAGGACAGTCGAGATC
OASTL	GCTGGAACTGGTGGAACGGTTAG	ACCTGGTTCTCCTCCGCTAAGTAC
Actin	CTGTTCCAATCTACGAGGGTTTCT	GCTCGGCTGTGGTGGTGAA

### Statistical analysis

Three biological replicates were performed for all assays and the results were represented as mean values.SPSS 19.0 (SPSS Institute Inc.,USA) and Tukey’s test were used for pairwise comparisons between treatments and to perform multiple comparisons using Duncan’s new multiple range test (P < 0.05).

## Results

### Effect of sulfur on morphological indexes of cabbage

As shown in Figs 1 and 2, during the growth process of cabbage, sulfur deficiency seriously affected the growth and development of cabbage. The plant height and stem diameter of sulfur treatment were higher than those of sulfur-free treatment (except for the plant height at mature stage), and the difference was significant. In seedling stage, plant height and stem diameter of +S treatment were 34.3% and 55.9% higher than those of -S treatment, respectively. In rosette stage, plant height and stem diameter of +S treatment were 4.3% and 29% higher than those of -S treatment, respectively. In heading stage, plant height and stem diameter of +S treatment were 15.1% and 15.6% higher than those of -S treatment, respectively. In mature stage, plant height and stem diameter of +S treatment were 2.57% and 9.9% higher than those of -S treatment, respectively. In summary, sulfur deficiency at the seedling stage had the greatest impact on plant height and stem diameter of cabbage.

**Fig 1:**
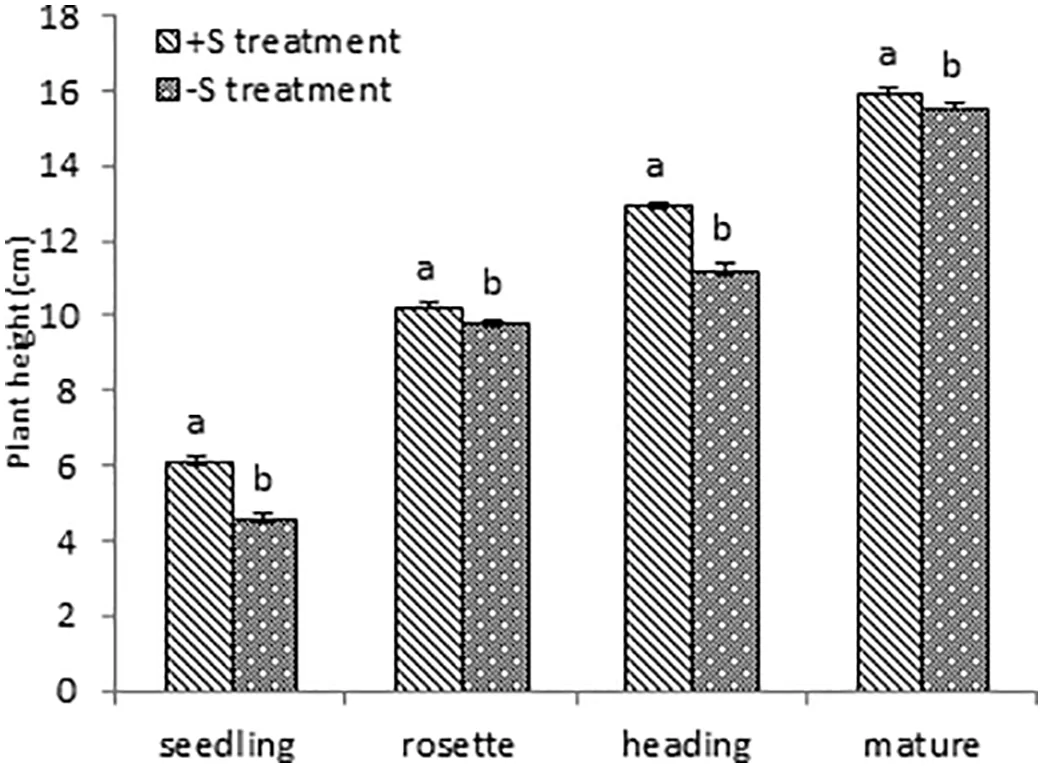
Effects of +S treatment and -S treatment on plant height of cabbage during different growth stages.

**Fig 2:**
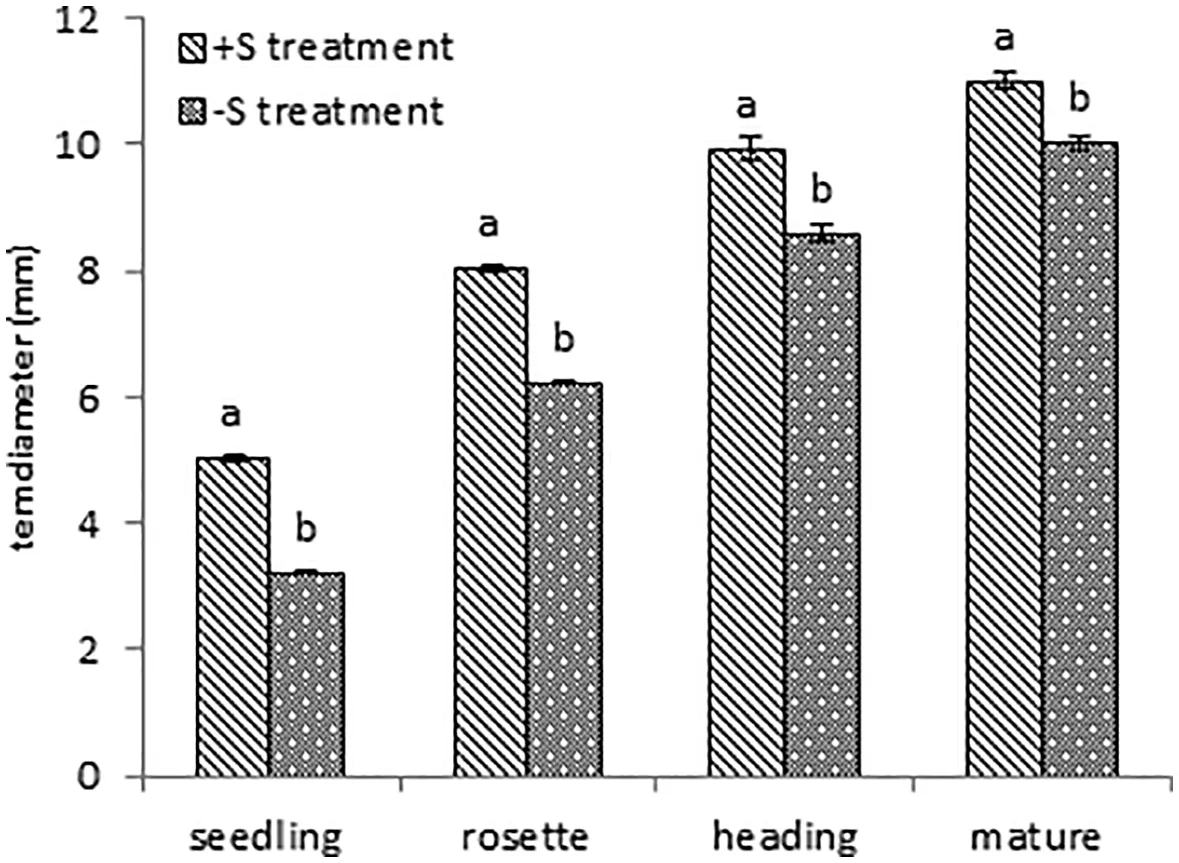
Effects of +S treatment and -S treatment on stem diameter of cabbage during different growth stages. Note: Different lowercase letters indicate significant differences (p < 0.05) among sulfur treatments during the same growth stage.

### Effects of sulfur on dry and fresh weight and root dry matter partitioning rate of cabbage

As shown in Fig 2, Fig 3, Fig 4, Fig 5, Fig 6, Fig 7, Fig 8 +S treatment significantly increased the fresh weight and dry weight of the shoot. The fresh weight of shoot under +S treatment increased by 90.9%, 43.6%, 19.2%, and 15.4% compared to that under -S treatment at seedling, rosette, heading, and mature stages, respectively; The aboveground dry weight increased by 51%, 27.8%, 15.7%, and 14.2%, respectively. The changes of underground dry/fresh weight and aboveground dry/fresh weight were contrary to each other. The underground dry/fresh weight of -S treatment was higher than that of +S treatment, and the underground fresh weight of +S treatment decreased by 21.2%, 20.62%, 9.1%, and 0.6% compared to that of -S treatment at seedling stage, rosette stage, heading stage, and mature stage, respectively; The underground dry weight increased by 0.7%, 21.1%, 0.8%, and 3.7%, respectively. From the perspective of dry matter allocation rate, the root dry matter allocation rate at the rosette and heading stages was the highest at the rosette and heading stages, reaching 15.47% and 13.09% under +S treatment; 10.15% and 10.4% for -S processing.

**Fig 3:**
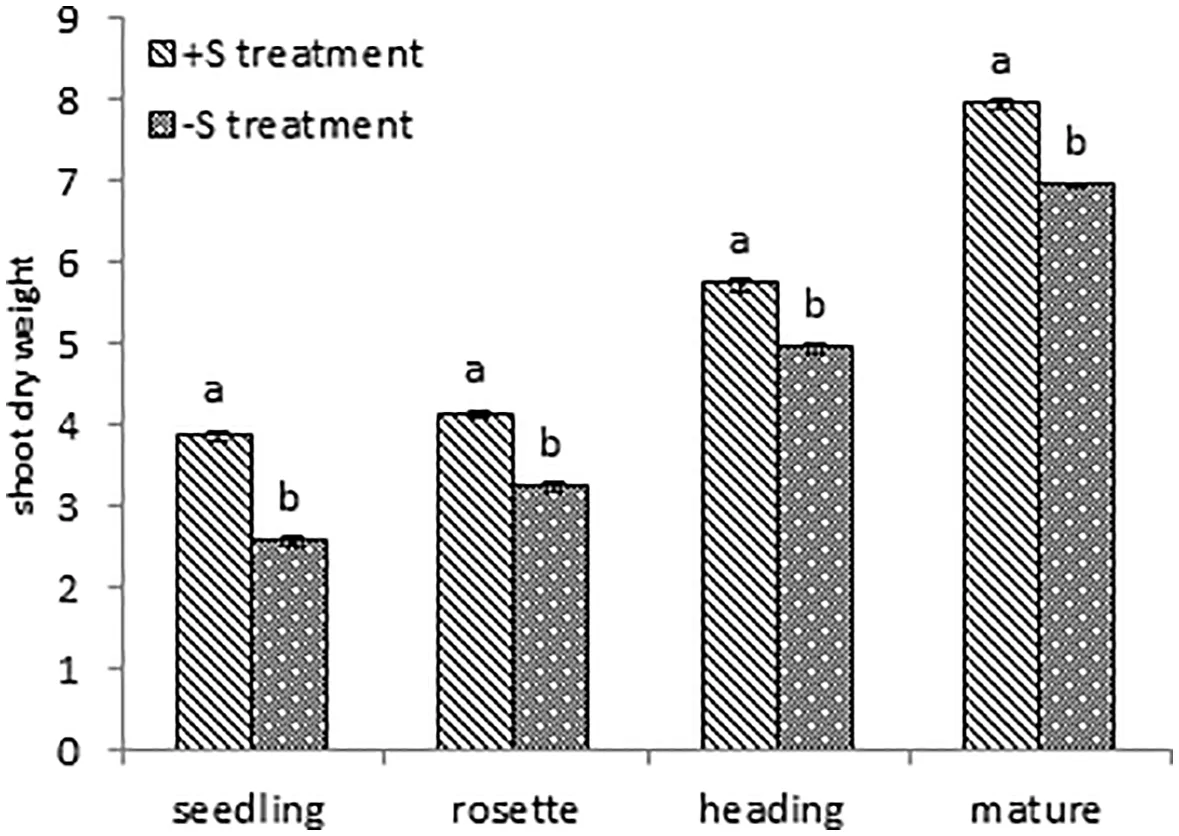
Effects of +S treatment and -S treatment on shoot dry weight of cabbage during different growth stages.

**Fig 4:**
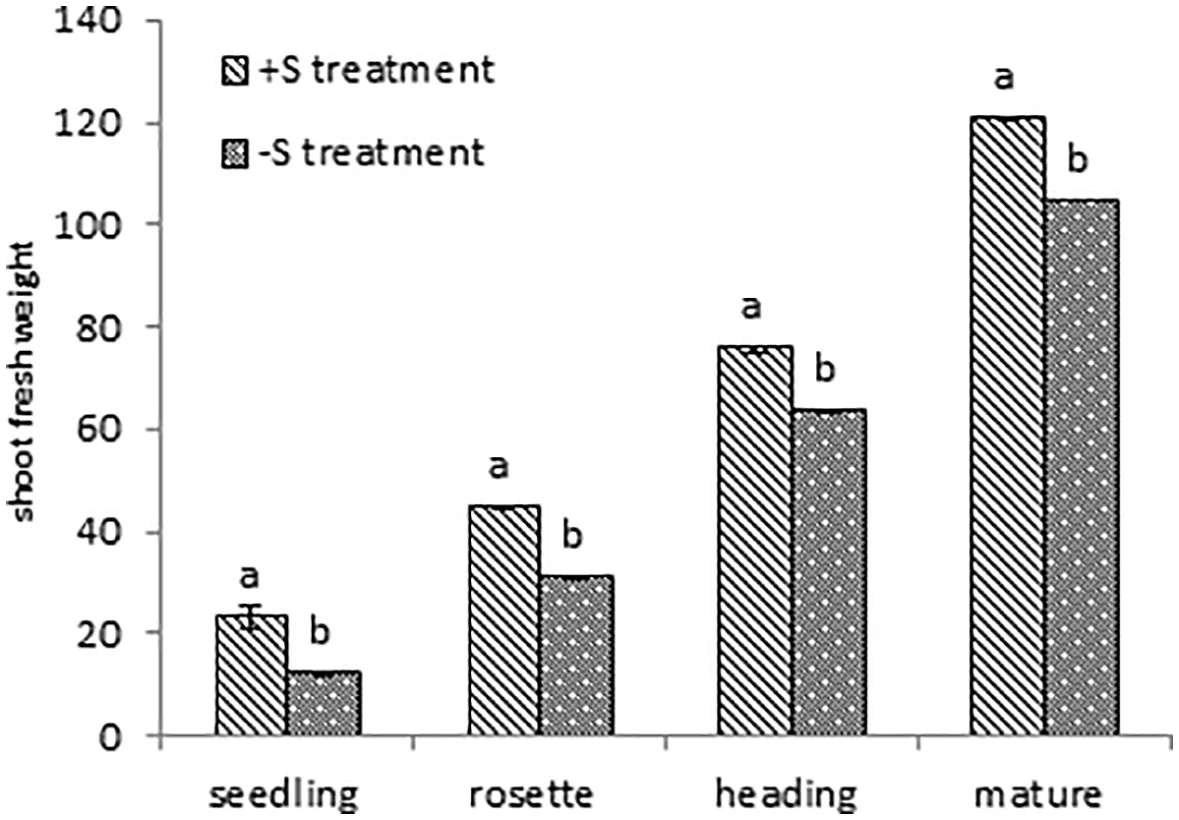
Effects of +S treatment and -S treatment on shoot fresh weight of cabbage during different growth stages.

**Fig 5:**
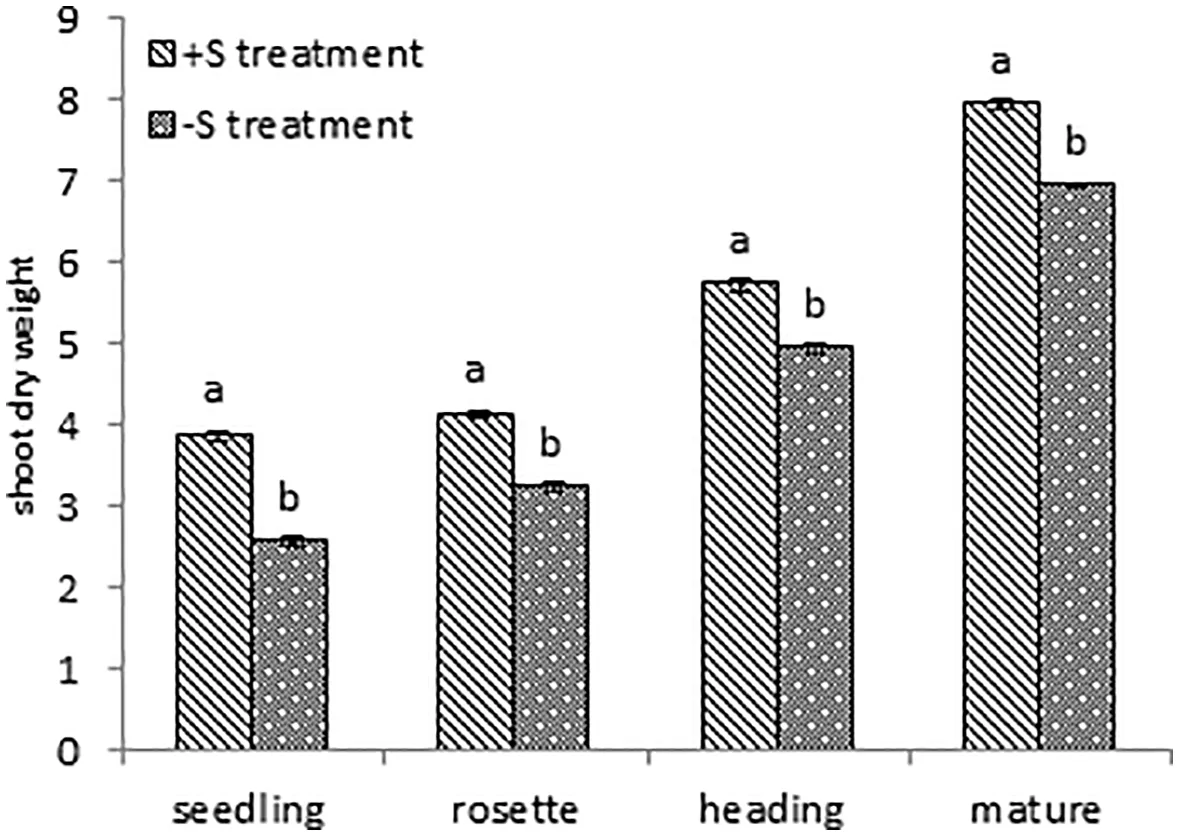
Effects of +S treatment and -S treatment on root dry weight of cabbage during different growth stages.

**Fig 6:**
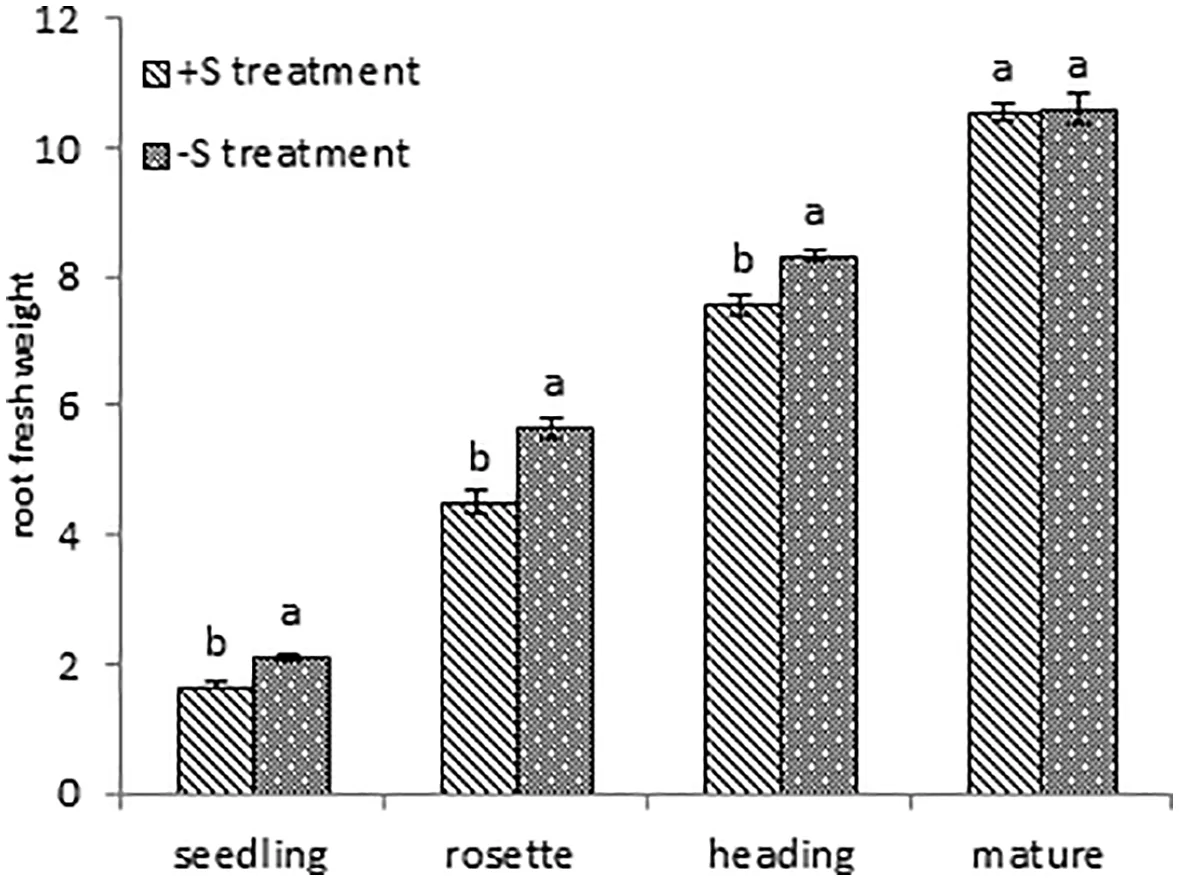
Effects of +S treatment and -S treatment on root fresh weight of cabbage during different growth stages.

**Fig 7:**
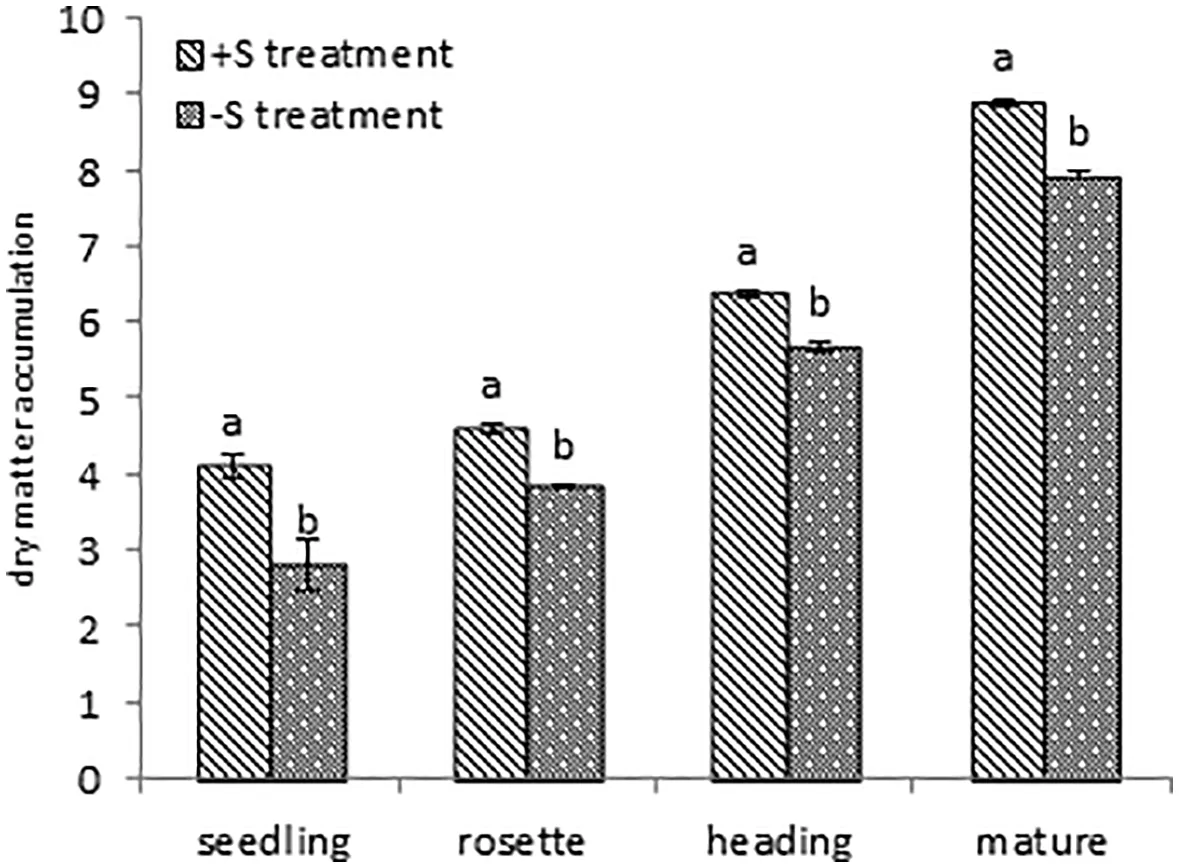
Effects of +S treatment and -S treatment on plant dry matter accumulation of cabbage during different growth stages.

**Fig 8:**
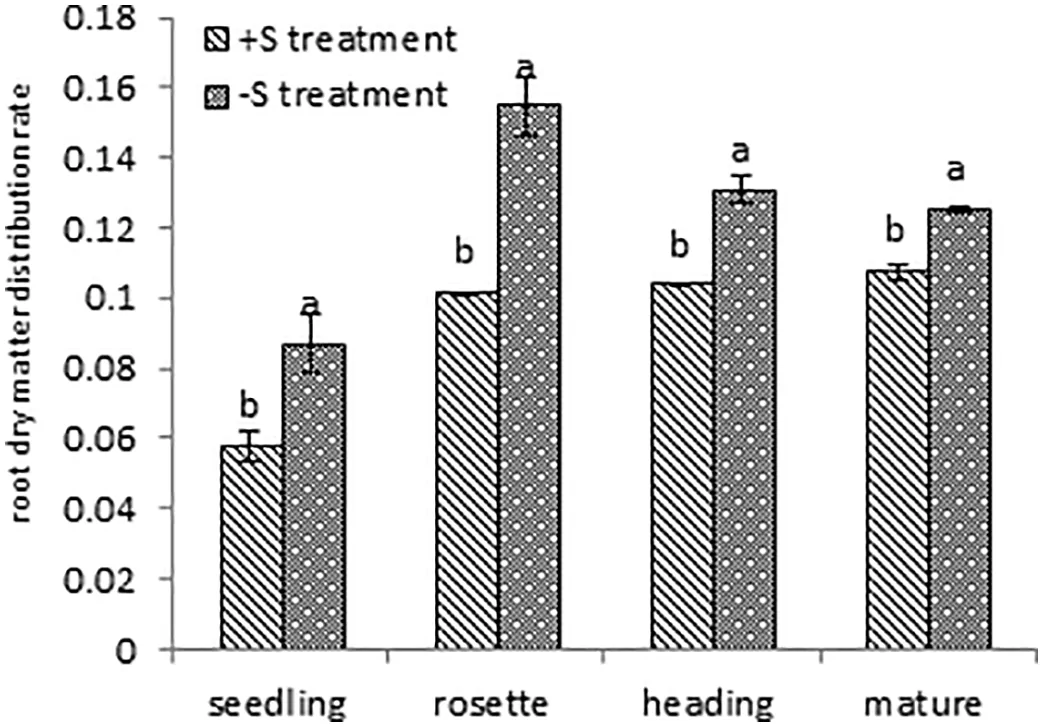
Effects of +S treatment and -S treatment on root dry matter distribution rate of cabbage during different growth stages. Note: Different lowercase letters indicate significant differences (p < 0.05) among sulfur treatments during the same growth stage.

### Effect of sulfur on photosynthetic pigments

As shown in Table 1, +S and -S treatments had no significant effect on the total chlorophyll content, chlorophyll a, chlorophyll b and carotenoid contents in cabbage leaves. However, the contents of total chlorophyll, chlorophyll a, chlorophyll b and carotenoid in +S treatment were higher than those in -S treatment. Total chlorophyll content increased by 39.47% at seedling stage, which was 2.1%, 5.7% and 21.9% higher than that at the rosette stage, heading stage and mature stage, respectively, with the largest increase of 44.9%. Chlorophyll-a and chlorophyll-b increased by 24.0% and 15.5%, respectively, at the heading stage. Carotenoids reached the highest level at the heading stage, where the increase rate was also the highest, at 34.9%.

**Table 1:** Chlorophylla, Chlorophyllb, and Total chlorophyllcontent.

Treatment	Growth stage	Content of chlorophyll a	Content of chlorophyll b	Total chlorophyll content	carotenoid content
+S	Seedling	0.73 ± 0.02a	0.21 ± 0.01a	0.94 ± 0.01a	0.30 ± 0.01a
	Rosette	0.83 ± 0.02a	0.21 ± 0.08a	1.05 ± 0.07a	0.32 ± 0.01a
	Heading	0.74 ± 0.03a	0.27 ± 0.02a	1.01 ± 0.01a	0.30 ± 0.00a
	Mature	0.70 ± 0.01a	0.24 ± 0.01a	0.94 ± 0.01a	0.25 ± 0.00a
-S	Seedling	0.56 ± 0.05a	0.12 ± 0.01a	0.67 ± 0.06b	0.23 ± 0.01a
	Rosette	0.67 ± 0.02b	0.08 ± 0.02b	0.76 ± 0.034b	0.23 ± 0.01ab
	Heading	0.62 ± 0.03b	0.12 ± 0.02b	0.74 ± 0.05b	0.21 ± 0.01a
	Mature	0.58 ± 0.01b	0.13 ± 0.03b	0.71 ± 0.03b	0.21 ± 0.01a

*μmol·g^-1^ FW is the unit for the total chlorophyll content, chlorophyll a, chlorophyll b and carotenoid content of cabbage leaves.

Significant comparison was made by sulfur/sulfur free treatment in different growth stages (p < 0.05).

As shown in Fig 9 that the obvious red area of +S treatment leaves was larger than that of -S treatment, and the red color was deeper, indicating that the chlorophyll content in +S treatment leaves was higher than that in -S treatment leaves. This is consistent with the results of chlorophyll content determined by spectrophotometer.

**Fig 9:**
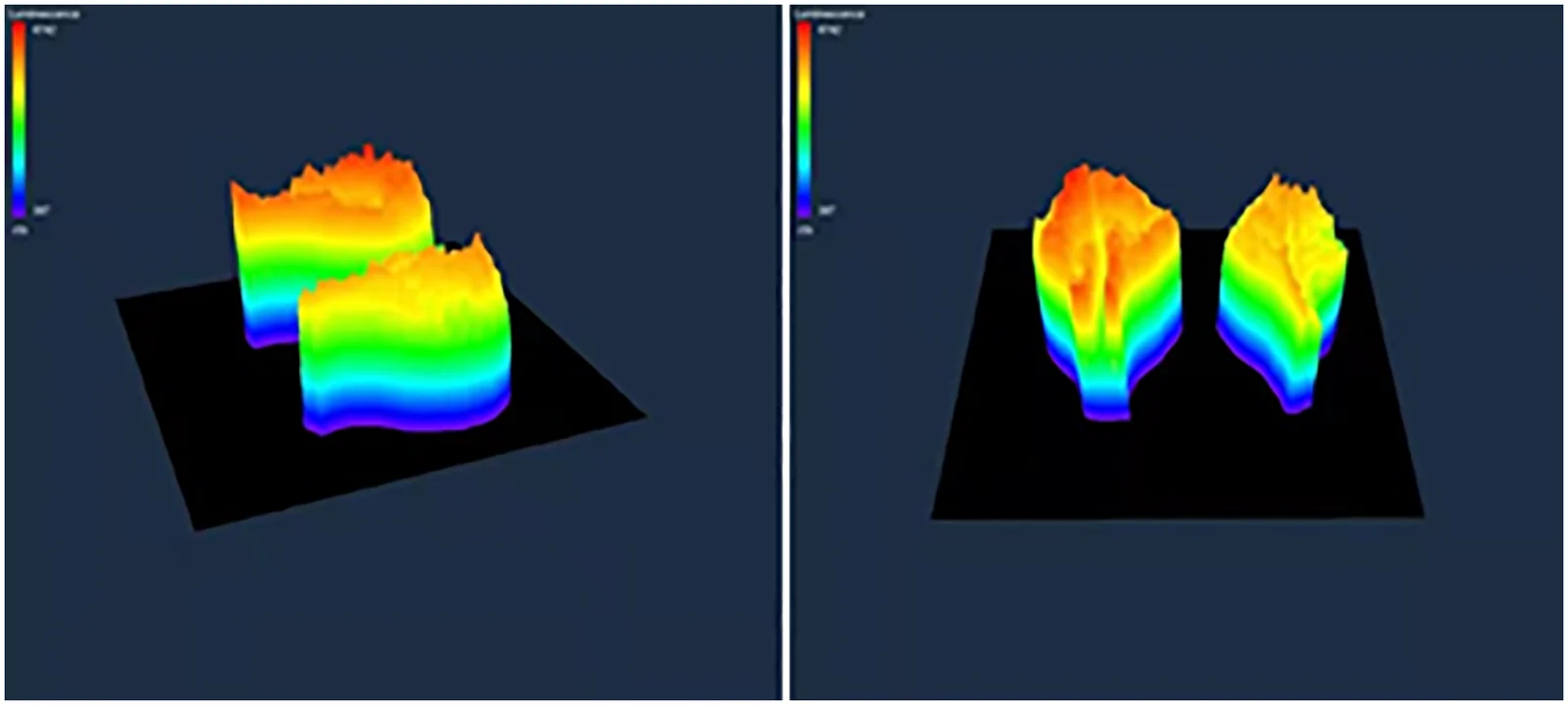
Effects of Sulfur on Chlorophyll Content of cabbage Leaves. Note: Sulfur treated on the right cabbage leaves and free on the left. It showed that the more chlorophyll content added, the more and redder areas came out.

### Effect of sulfur on gas excage parameters

AS shown in Table 2, the variation trends of net photosynthetic rate (Pn), stomatal conductance (Gs) and transpiration rate (E) were basically the same after sulfur application. The net photosynthetic rate, stomatal conductance and transpiration rate reached the maximum at the heading stage, heading stage> mature stage> rosette stage> seedling stage (except GS rosette stage and expansion stage), with significant differences among stages.Compared with the -S treatment, Pn, Gs, and E of +S treatment increased by 4%, 8% and 11%, respectively. The intercellular carbon dioxide concentration (Ci) exhibited a similiar variation trend to that of Pn; Ci was the highest and Pn reached the maximum at the heading stage, and Ci of +S treatment was 15% higher than that of -S treatment. These results indicate that +S treatment could effectively improved the photosynthesis of cabbage leaves, while -S treatment did not affect the trend of photosynthesis increasing first and then decreasing during cabbage growth.

**Table 2:** Effects of sulfur on gas excage parameters.

Treatment	Growth stage	IntercellularCO_2_ concentration ((μmol·m⁻^2^·g⁻^1^)μL·L⁻^1^)dioxide concentration	transpiration rate (μmol·m⁻^2^·g⁻^1^)	stomatal conductance (μmol·m⁻^2^·g⁻^1^)	net photosynthetic rate (μmol·m⁻^2^·g⁻^1^)
+S	Seedling	448.00 ± 5.51a	5.30 ± 0.06a	366.00 ± 2.08a	21.10 ± 0.44a
	Rosette	402.33 ± 1.45a	8.37 ± 0.12a	401.67 ± 2.67a	25.10 ± 0.15a
	Heading	355.00 ± 2.08a	10.23 ± 0.03a	442.00 ± 3.79a	28.27 ± 0.19a
	Mature	379.00 ± 1.73a	8.50 ± 0.10a	401.67 ± 1.86a	25.57 ± 0.18a
-S	Seedling	391.67 ± 3.84a	5.17 ± 0.13a	349.00 ± 1.73a	18.40 ± 0.15a
	Rosette	370.67 ± 2.03b	7.87 ± 0.15a	369.00 ± 1.16b	23.20 ± 0.21a
	Heading	308.00 ± 1.73b	9.87 ± 0.12a	407.67 ± 1.45b	25.43 ± 0.09b
	Mature	353.00 ± 2.08b	8.37 ± 0.15a	389.33 ± 0.88ab	23.80 ± 0.17b

Significant comparison was made by sulfur/sulfur free treatment in different growth stages (p < 0.05).

### Effect of sulfur on chlorophyll fluorescence parameters

The chlorophyll fluorescence parameters of each treatment were measured to asses the effect of sulfur treatment on PSII photoinhibition (Table 3). The results showed that Y(II)/PSII, fv/fm, NPQ and qP decreased at each growth period under -S treatment, indicating that the proportion of light energy absorbed by PSII antenna pigments participating in chemical reactions decreased under -S treatment. The actual photochemical efficiency of PSII under S treatment was 18.7%, 20.7%, 19.7% and 24.4% higher than that under -S treatment at the seedling, rosette, heading and mature stages, respectively. The maximum photochemical efficiency of PSII was 5.2%, 3.1%, 13.6% and 13.5% higher in +S than in -S treatment at the corresponding growth stages. NPQ and qP in -S treatment were significantly lower than those in +S treatment, decreasing by 62% and 1.2%, respectively, at the seedling stage, indicating that -S treatment destroyed the photosynthetic electron transport chain.

**Table 3:** Effects of sulfur on chlororescence parameters.

Treatment	Growth stage	Y(II)/PSII	fv/fm	NPQ	qP
+S	Seedling	0.65 ± 0.00a	0.757 ± 0.00a	0.32 ± 0.02a	0.97 ± 0.00a
	Rosette	0.66 ± 0.00a	0.76 ± 0.00a	0.74 ± 0.00a	0.94 ± 0.00a
	Heading	0.68 ± 0.00a	0.835 ± 0.01a	0.79 ± 0.00a	0.89 ± 0.00a
	Mature	0.57 ± 0.01a	0.77 ± 0.00a	0.69 ± 0.00a	0.78 ± 0.014a
-S	Seedling	0.55 ± 0.01b	0.71 ± 0.00a	0.12 ± .02b	0.95 ± 0.01a
	Rosette	0.55 ± 0.01b	0.73 ± 0.00a	0.45 ± 0.00b	0.92 ± 0.01a
	Heading	0.57 ± 0.00b	0.73 ± 0.01a	0.75 ± 0.00b	0.95 ± 0.00b
	Mature	0.46 ± 0.01b	0.67 ± 0.00a	0.49 ± 0.00b	0.86 ± 0.01b

Significant comparison was made by sulfur/sulfur free treatment in different growth stages (p < 0.05).

### Effect of sulfur on antioxidant enzyme system

As shown in Figs 10–13, the activities of SOD, POD and CAT increased gradually throughnout the growth period of cabbage. Compared with -S treatment, + S treatment showed significantly different antioxidant enzyme activities at seedling, rosette, heading, and mature stages (except SOD and POD activities at heading stage). SOD activity increased by 40%, 34.7%, 10.7% and 20.7%; POD activity increased by 93.3%, 33.3%, 46.7% and 26.7%; CAT activity increased by 8.5%, 24.7%, 20.3% and 30.9%; and APX activity increased by 17.1%, 13.2%, 0% and 15.8%. The activities of SOD, POD and APX at the seedling stage were 15.3%, 180%, and 29.5% higher than those at the rosette stage, respectively. In conclusion, +S treatment significantly enhanced the activities of antioxidant enzymes in cabbage leaves, especially at the seedling stage.

**Fig 10:**
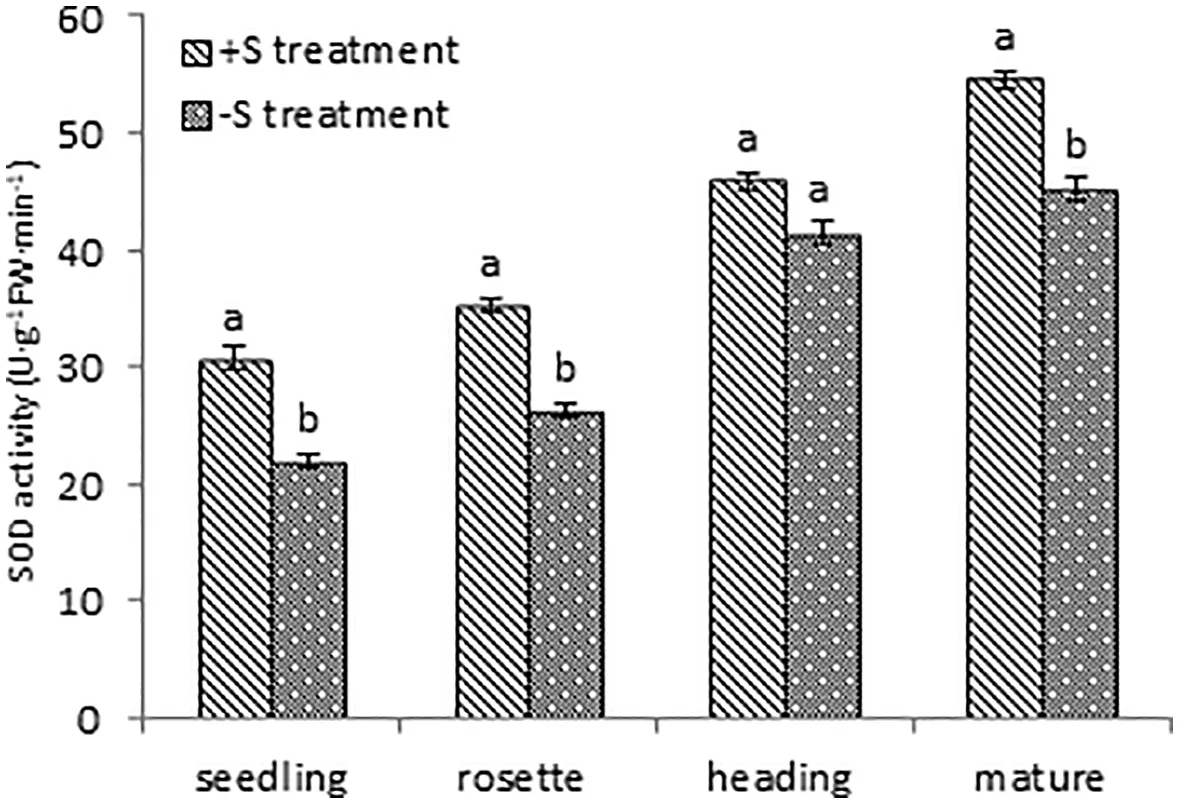
Effects of +S treatment and -S treatment on SOD activity of cabbage during different growth stages.

**Fig 11:**
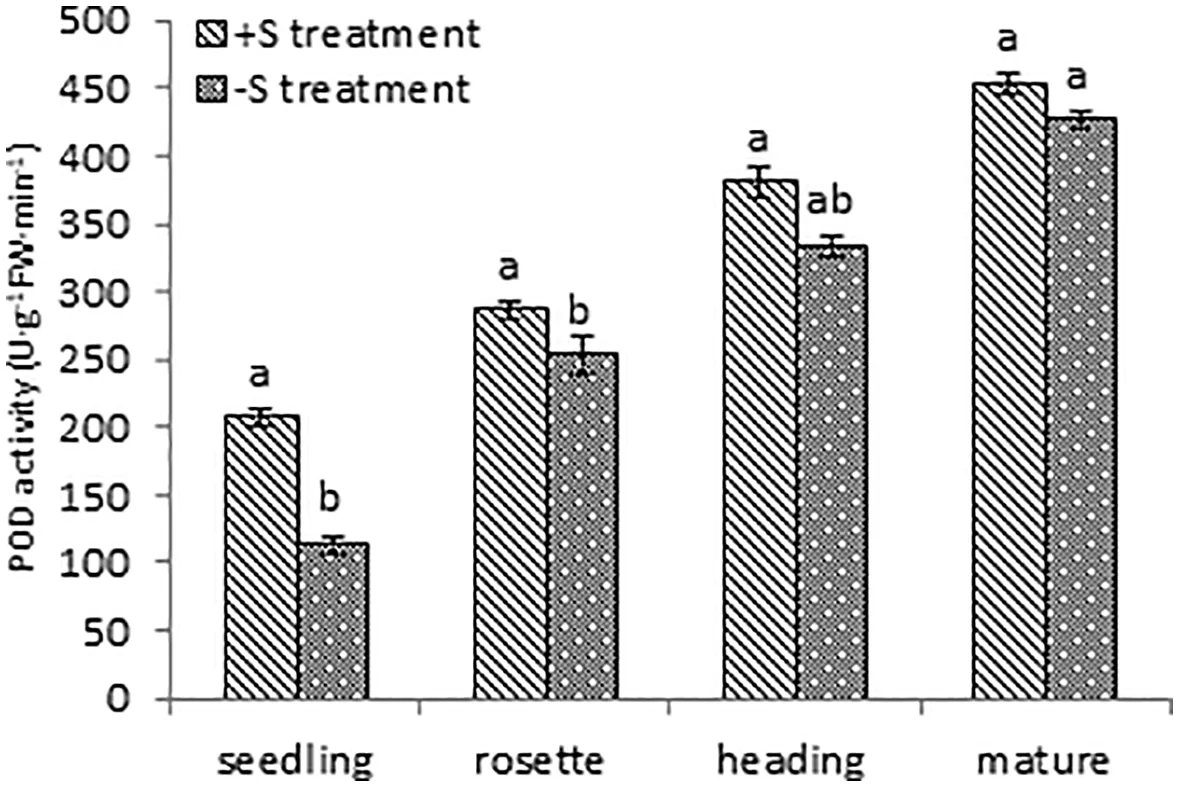
Effects of +S treatment and -S treatment on POD activity of cabbage during different growth stages.

**Fig 12:**
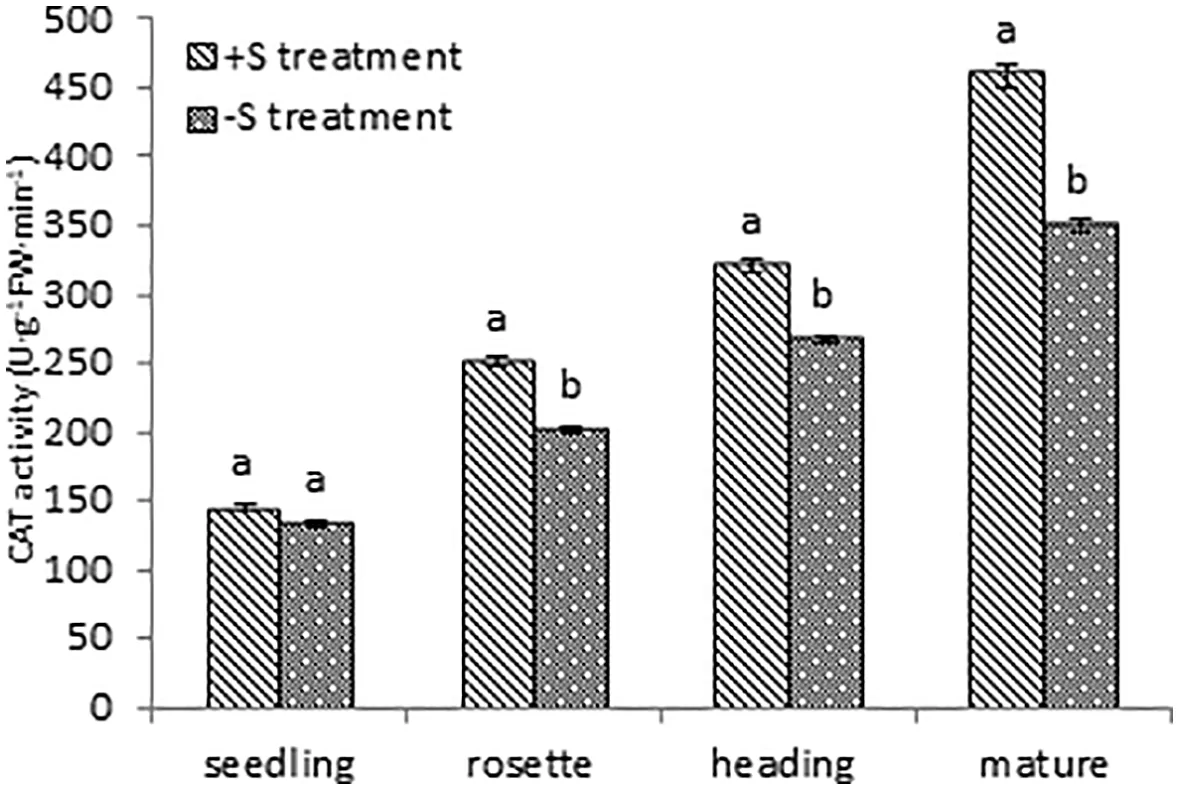
Effects of +S treatment and -S treatment on CAT activity of cabbage during different growth stages.

**Fig 13:**
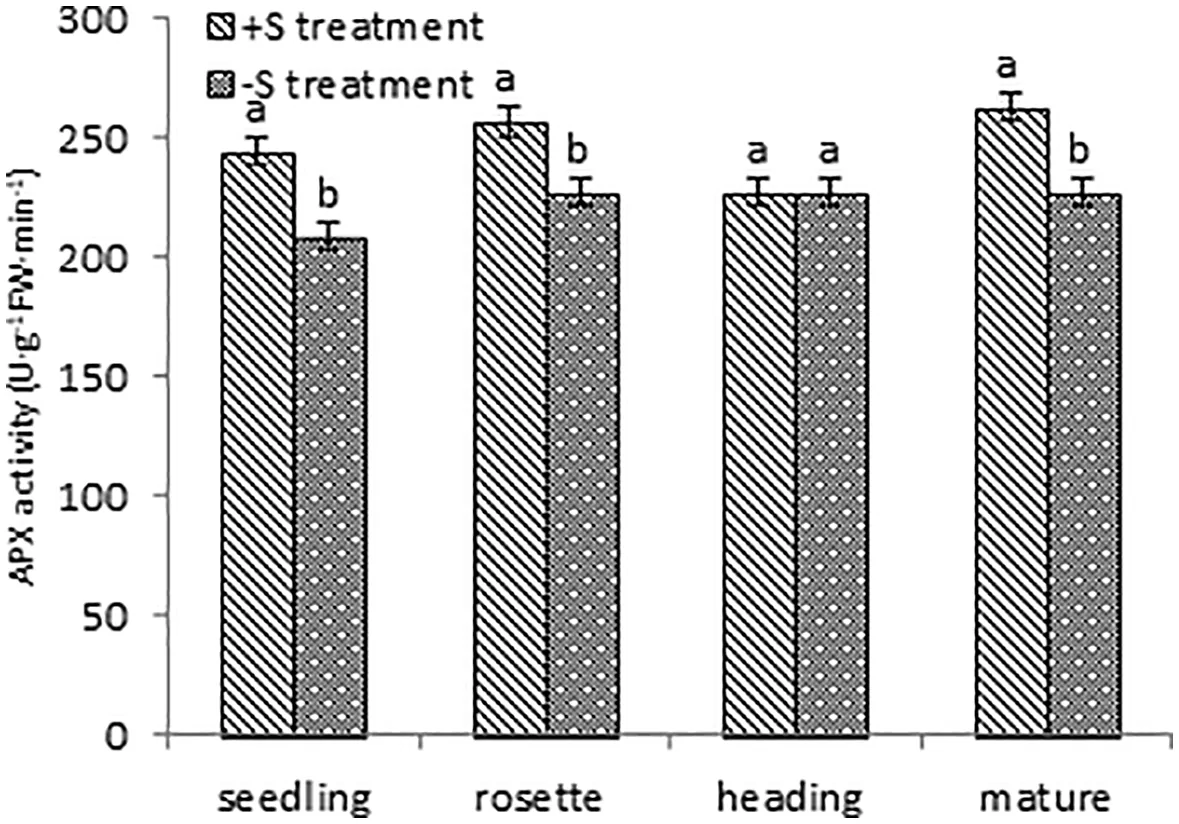
Effects of +S treatment and -S treatment on APX activity of cabbage during different growth stages. Note: Different lowercase letters indicate significant differences (p < 0.05) among sulfur treatments during the same growth stage.

### Effects of sulfur on glutathione circulation system and osmotic adjustment substances

Glutathione (GSH) is an important electron acceptor in plants. Its reduction to ASA is one of the important ways to scavenge oxygen free radicals in plants. Glutathione is also an important intermediate substance of glucosinolates, and its content directly influences the content of glucosinolates. As shown in Figs 14 and 15, GSH content in cabbage first increased and then decreased throughout the growth period under +S and -S treatments, reaching its maximum at the heading stage. The GSH content of +S and -S treatments increased by 9.1% and 11.9%, respectively, at the rosette stage. The GSH content of +S treatment was higher than that of -S treatment in the whole growth period of cabbage, and the difference was significant, which increased by 19.2%, 11.1%, 13.9% and 11.9% respectively in seedling, rosette, heading, and mature stages. Under +S treatment, GSSG exhibited the same trend as GSH throughout the growth period, and the content of GSSG was the highest at the heading stage.

**Fig 14:**
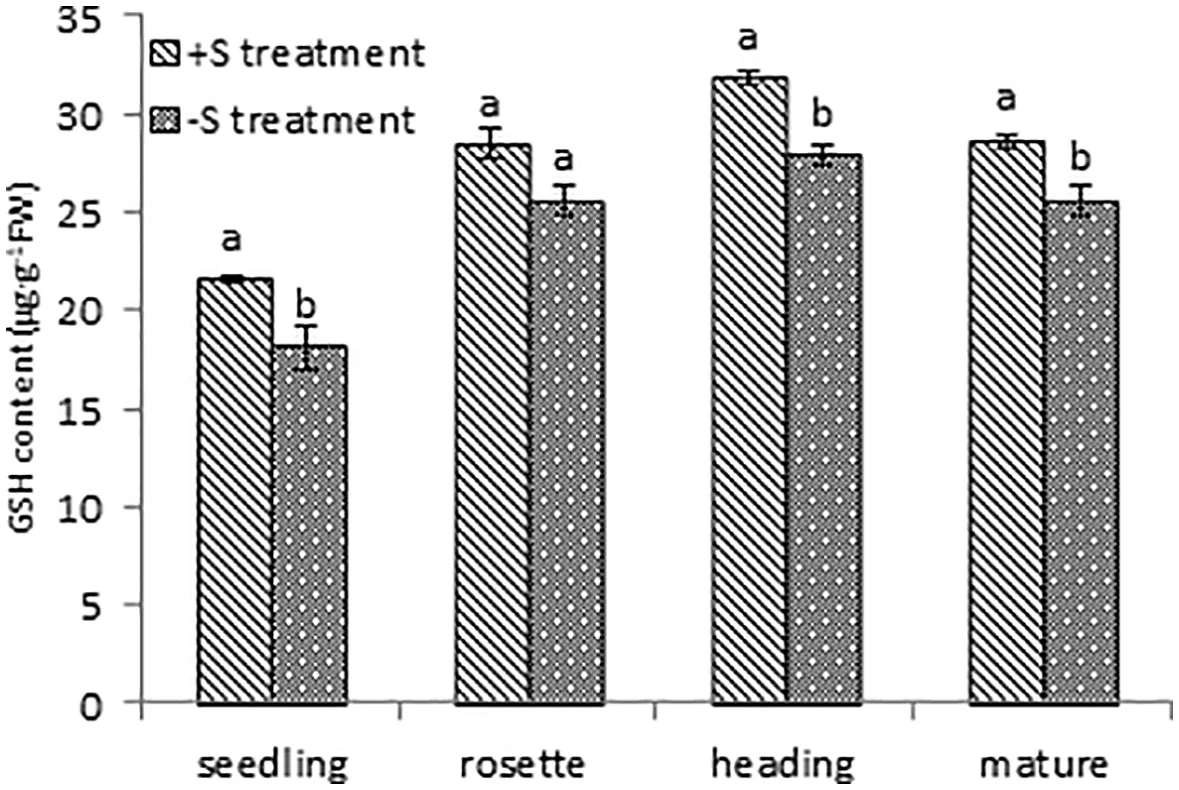
Effects of +S treatment and -S treatment on GSH content of cabbage during different growth stages.

**Fig 15:**
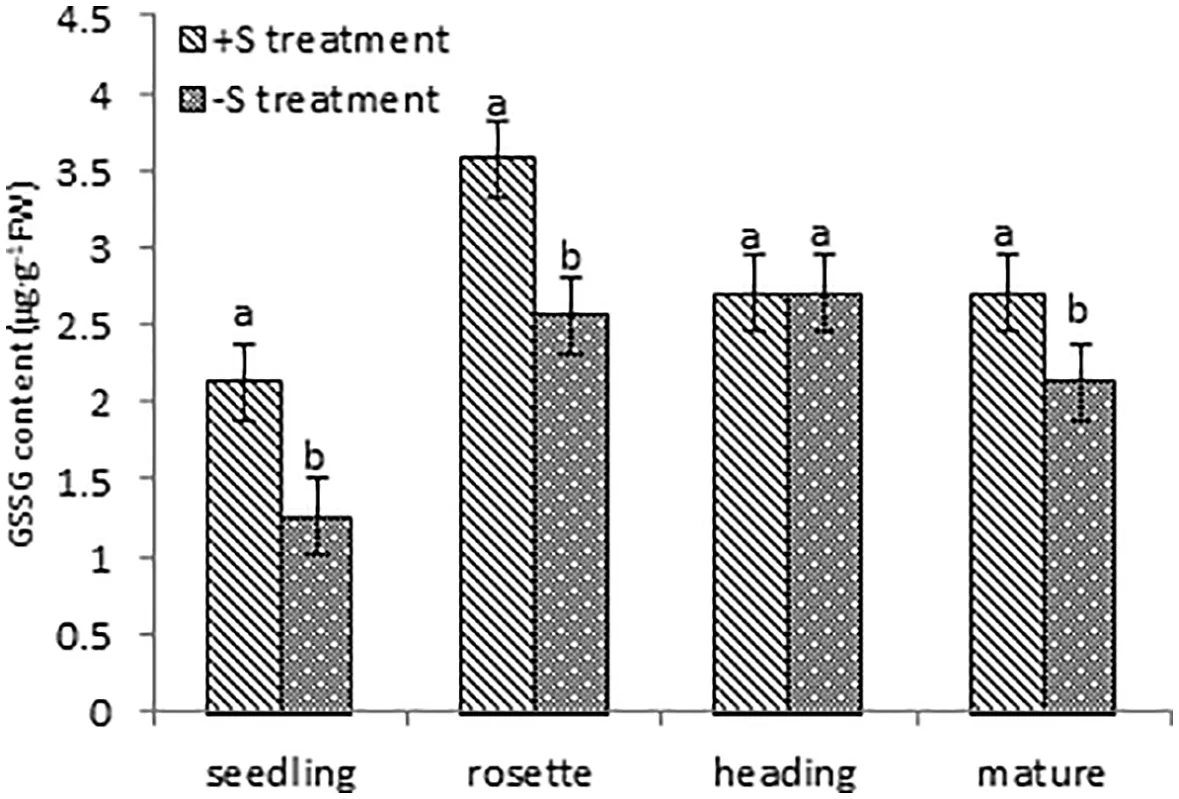
Effects of +S treatment and -S treatment on GSSG content of cabbage during different growth stages. Note: Different lowercase letters indicate significant differences (p < 0.05) among sulfur treatments during the same growth stage.

### Effect of sulfur on sulfur-metabolizing enzymes

From Figs 16–20, it can be seen that +S and -S treatments had different effects on the activities of sulfur metabolism enzymes in cabbage leaves. The activities of ATP sulfurylase (ATPS) and APS reductase (APR) were significantly increased by S treatment. ATPS activity under the + S treatment was 15.5%, 13.8%, 45.2% and 25.7% higher than those of -S treatment, respectively. APR activity increased by 35%, 24.8%, 67.3% and 63.1%, respectively. The activities of ATPS and APR increased initially and then decreased during cabbage growth, and reached the maximum at heading stage. The changes of serine acetyltransferase (SAT) and O-acetylserine transferase (OASTL) under +S and -S treatments were similar to those of ATPS and APR. At seedling stage, the activities of SAT and OASTL under +S treatment were significantly different from those under -S treatment.. The activities of SAT and OASTL under +S treatment were 1.07 and 1.38 times higher than those under +S treatment, respectively. In addition, sulfite reductase (SIR) activity increase by 14.8%, 16.5%, 17.0% and 25.9% at the seedling, rosette, heading, and mature stages, respectively.

**Fig 16:**
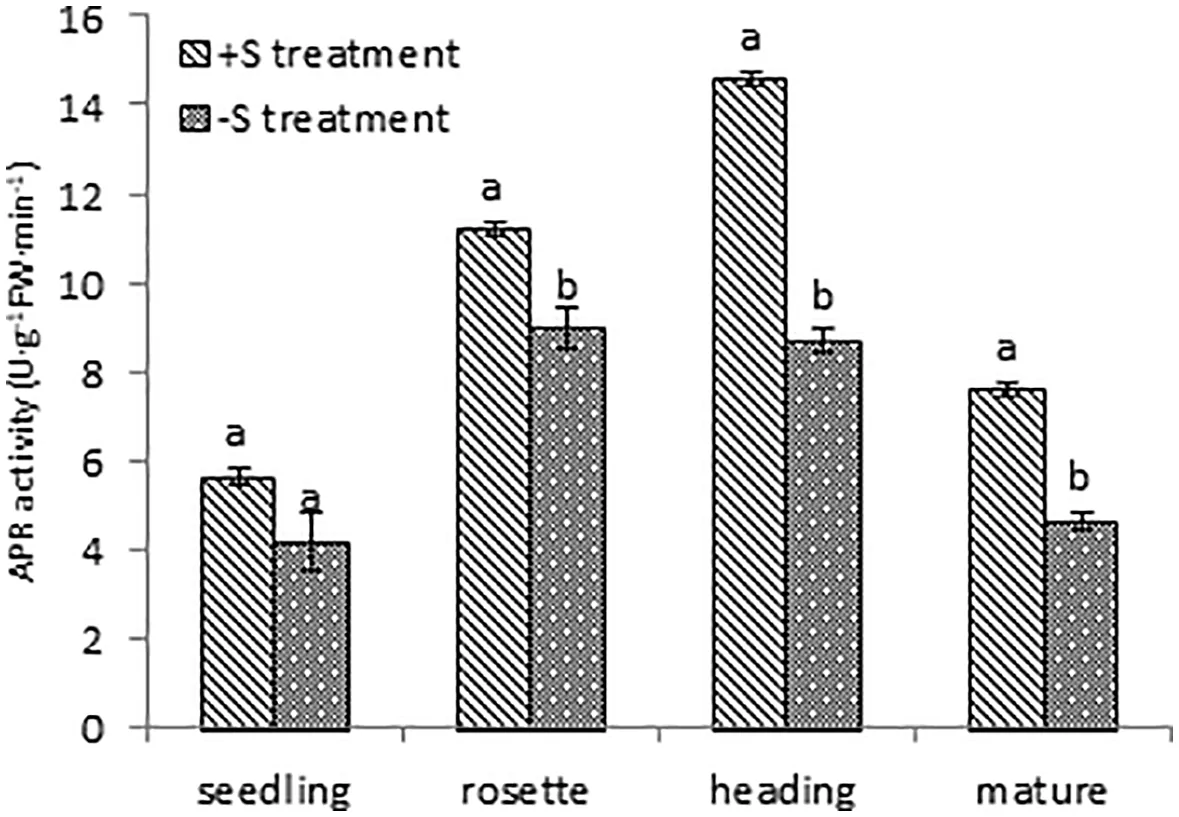
Effects of +S treatment and -S treatment on APR activity of cabbage during different growth stages.

**Fig 17:**
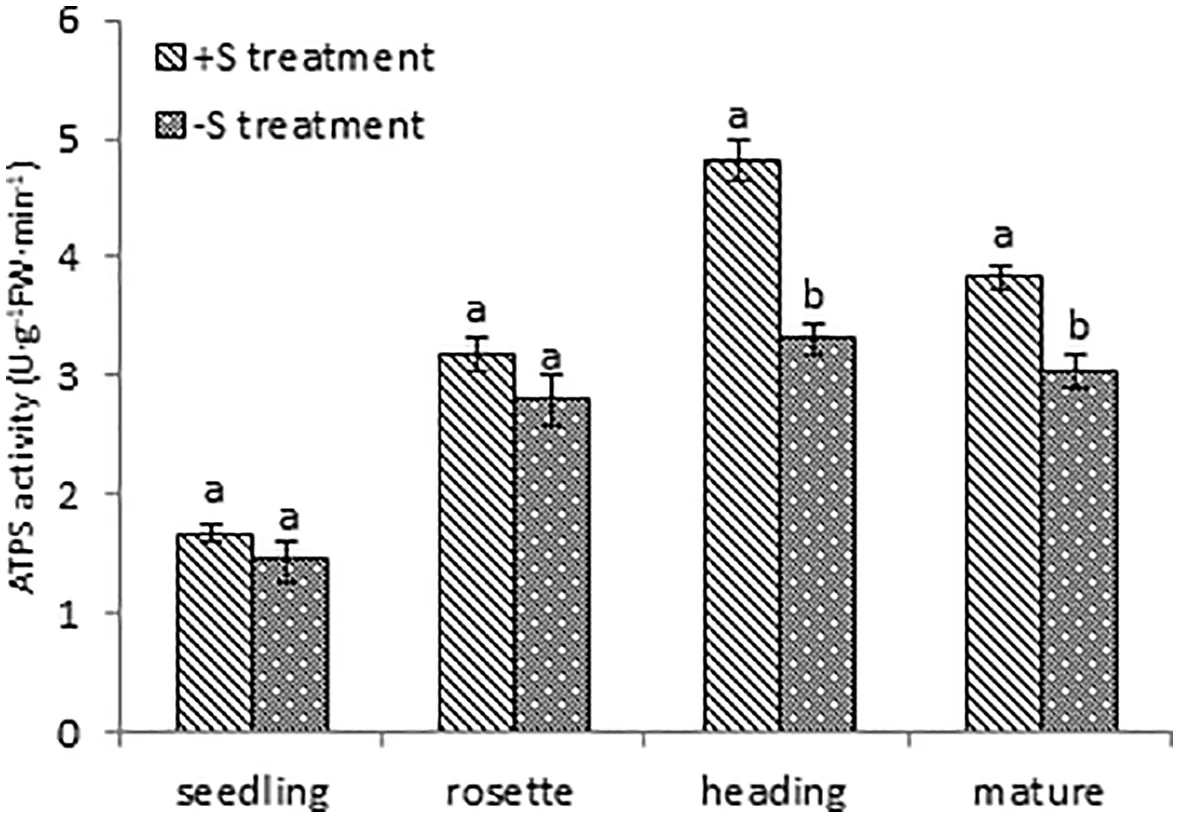
Effects of +S treatment and -S treatment on ATPS activity of cabbage during different growth stages.

**Fig 18:**
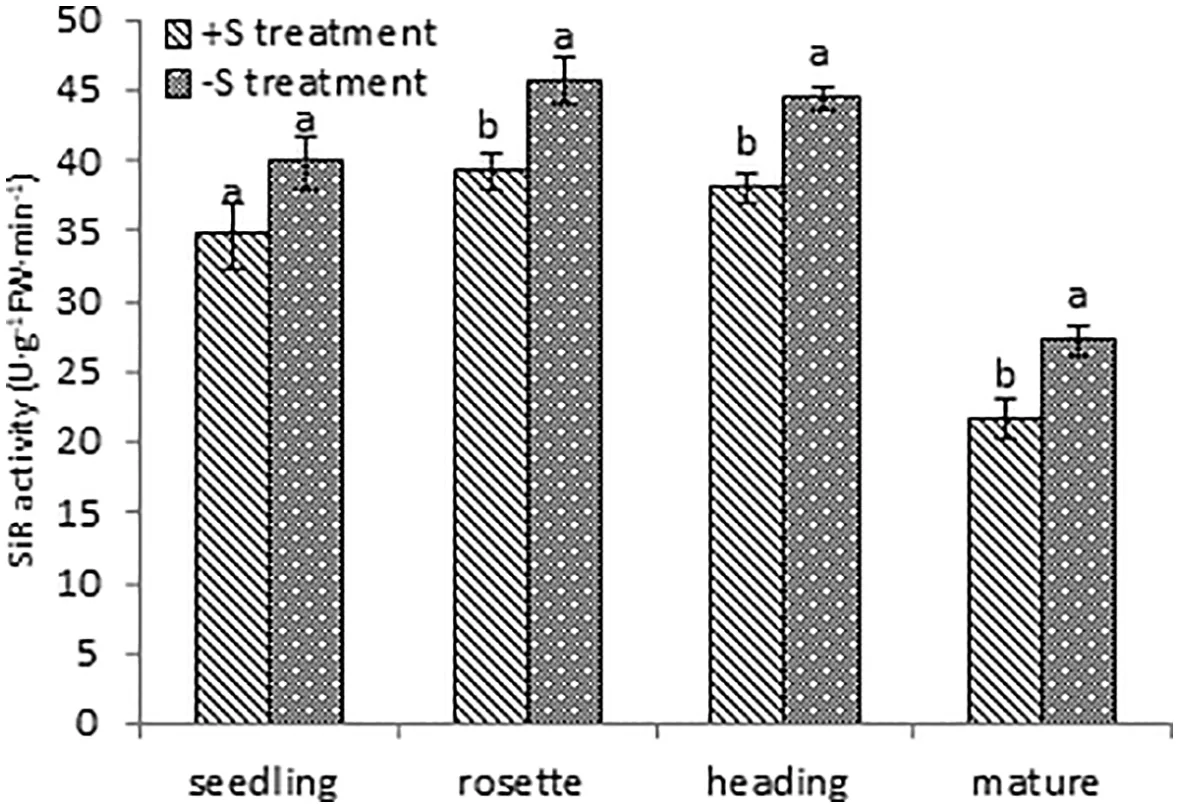
Effects of +S treatment and -S treatment on SiR activity of cabbage during different growth stages.

**Fig 19:**
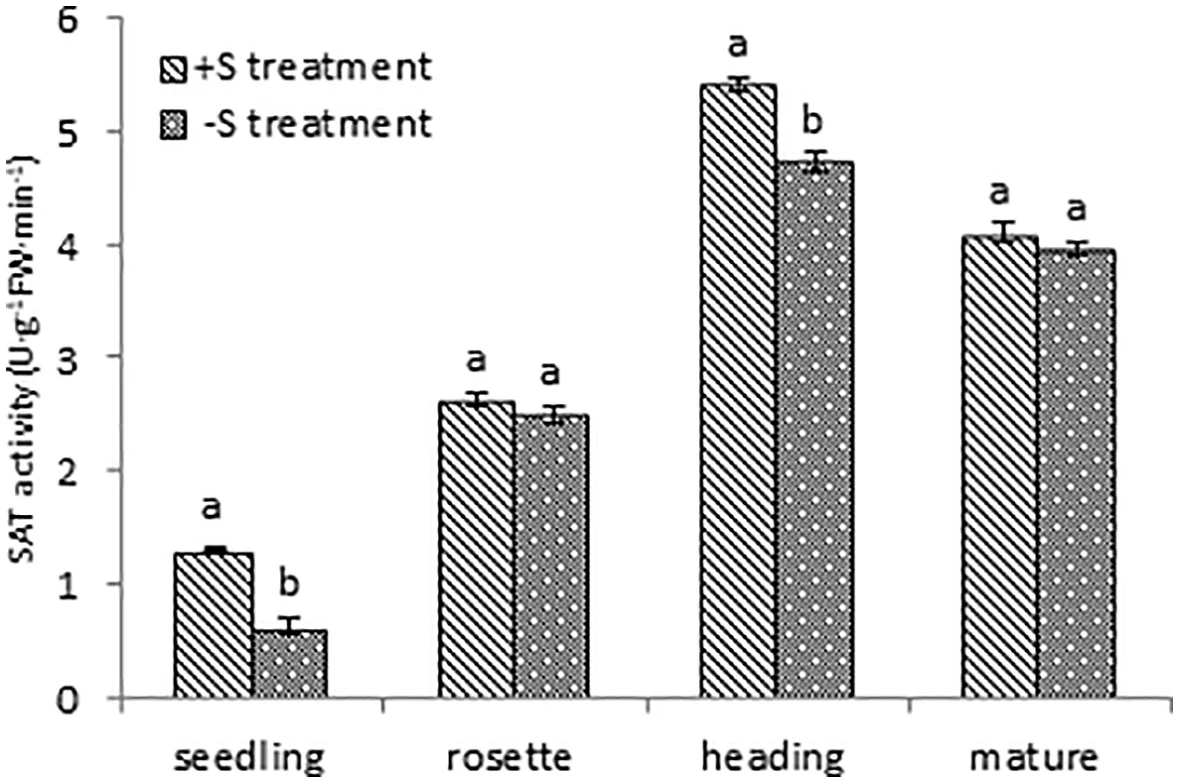
Effects of +S treatment and -S treatment on SAT activity of cabbage during different growth stages.

**Fig 20:**
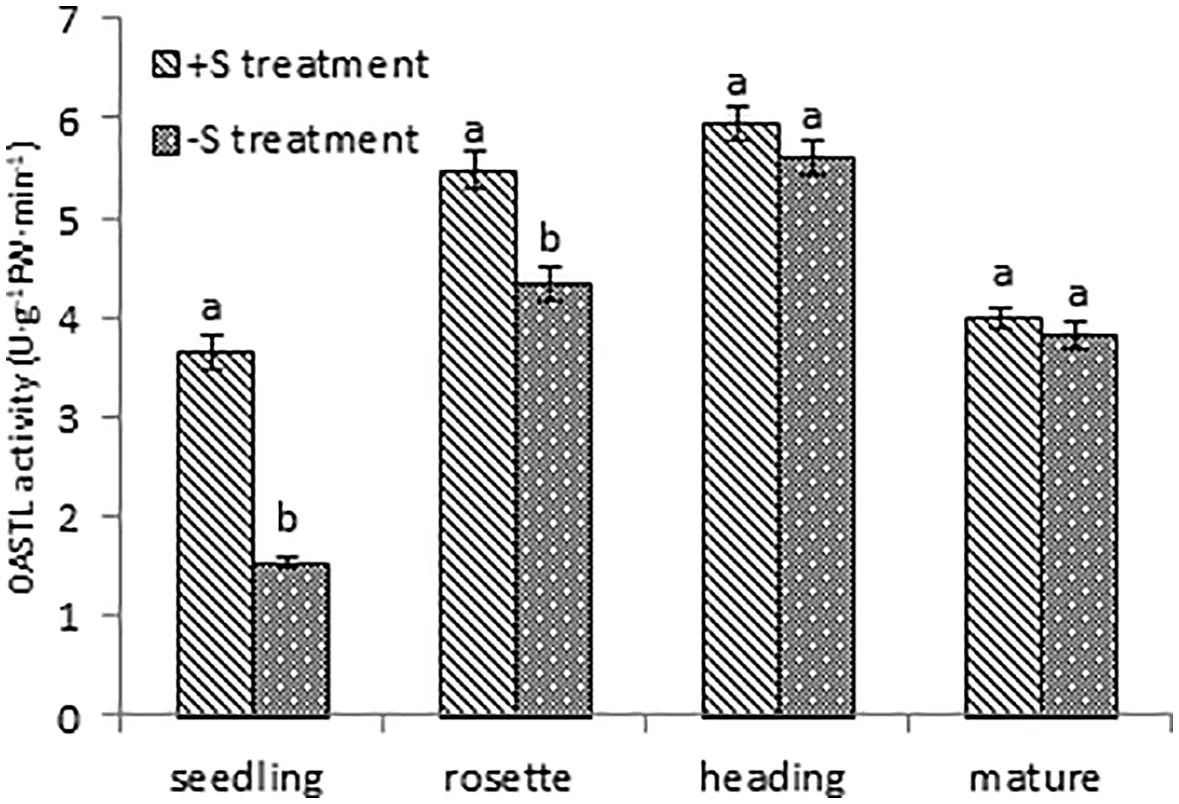
Effects of +S treatment and -S treatment on OASTL activity of cabbage during different growth stages. Note: Different lowercase letters indicate significant differences (p < 0.05) among sulfur treatments during the same growth stage.

### Effect of sulfur on nitrogen metabolism enzymes

Under the +S and -S treatments, the trend of nitrogen metabolism enzyme activity was different with the addition of sulfur (Figs 21–24). Nitrate reductase (NR) activity under -S treatment was higher than under +S treatment at the seedling, heading, and mature stages, but there was no significant difference under the same treatment. The activities of GOGAT and GS in +S treatment were 16.4% and 7.4% higher than those in -S treatment at the seedling stage, and with the highest activity observed throughout the entire growth period. Nitrite reductase (NIR) activity increased by 16.4%, 10.1%, 6.5% and 0.4% in the entire growth period. GS activity increased by 7.5%, 10%, 6.1% and 4.7% in the entire growth period.

**Fig 21:**
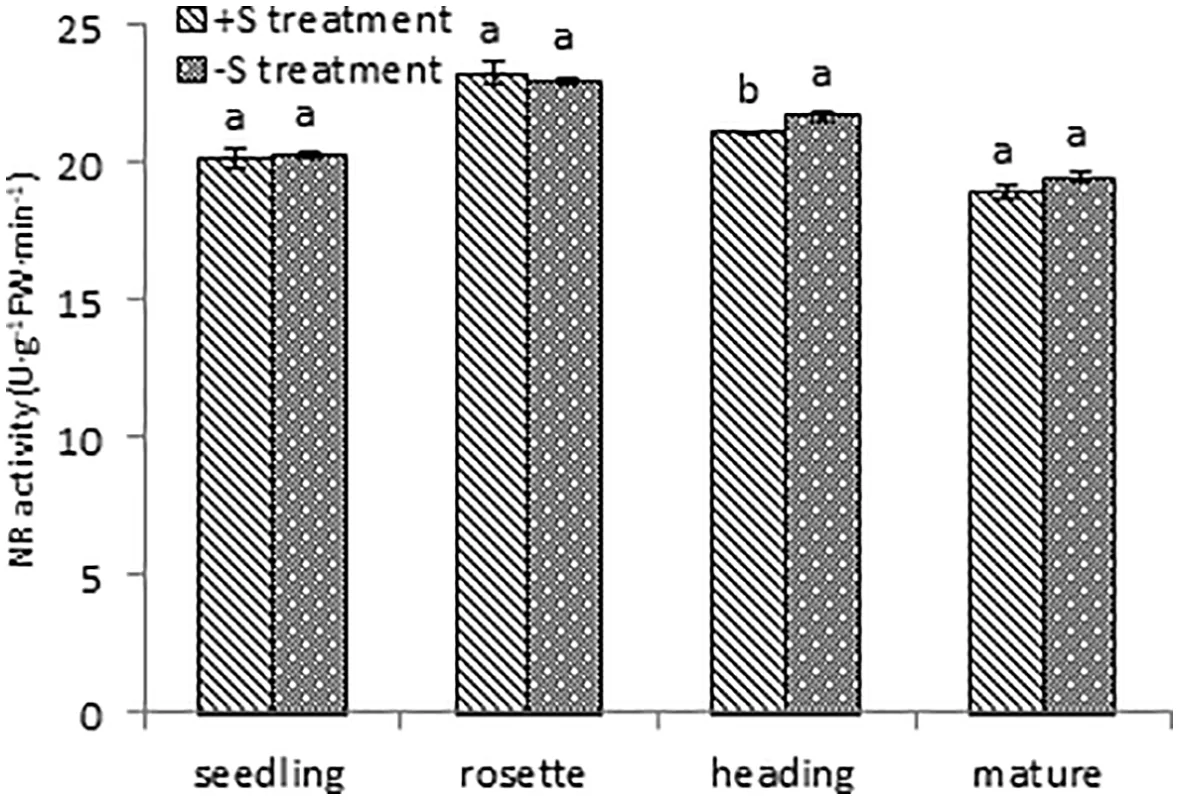
Effects of +S treatment and -S treatment on NR activity of cabbage during different growth stages.

**Fig 22:**
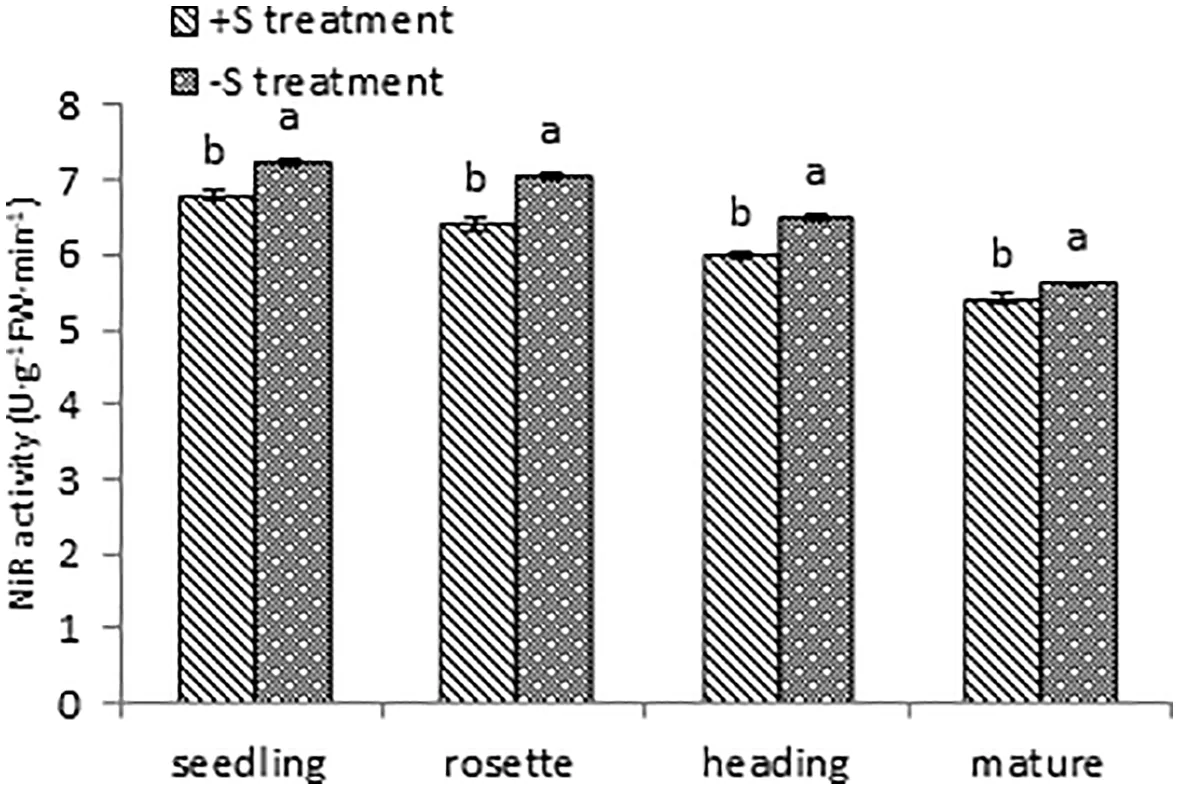
Effects of +S treatment and -S treatment on NiR activity of cabbage during different growth stages.

**Fig 23:**
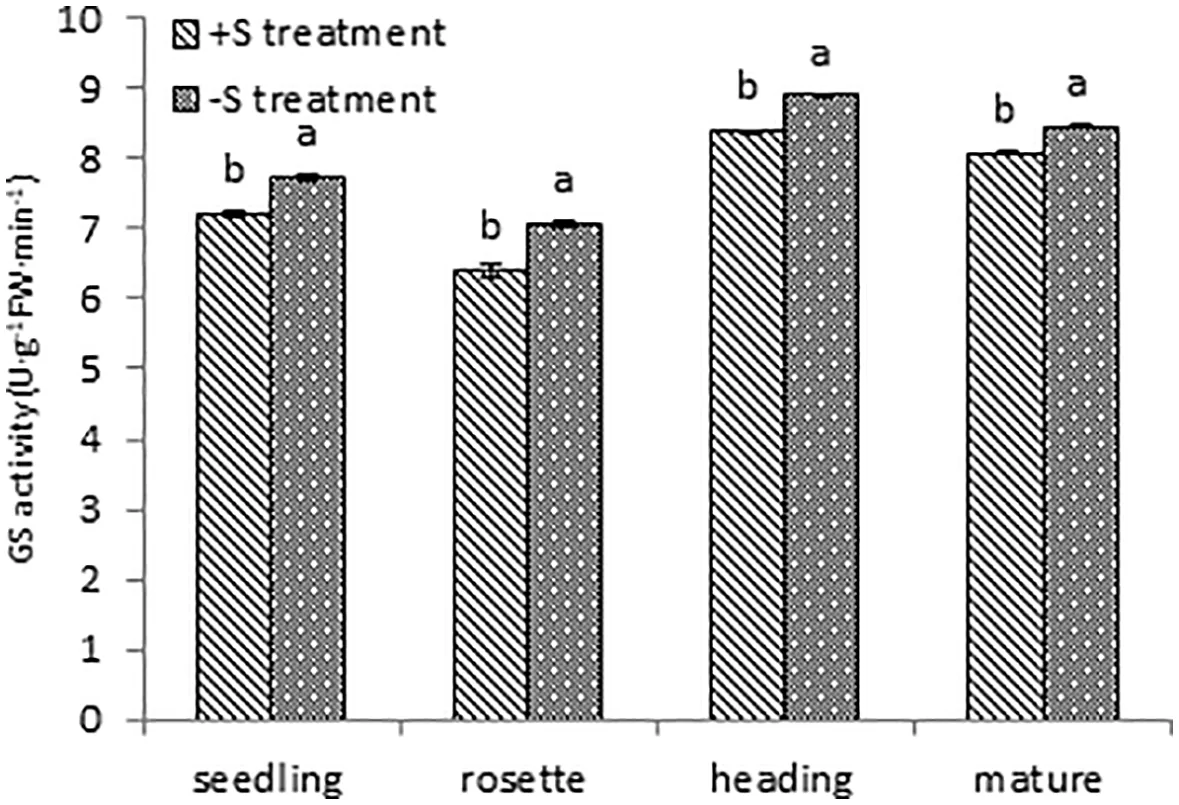
Effects of +S treatment and -S treatment on GS activity of cabbage during different growth stages.

**Fig 24:**
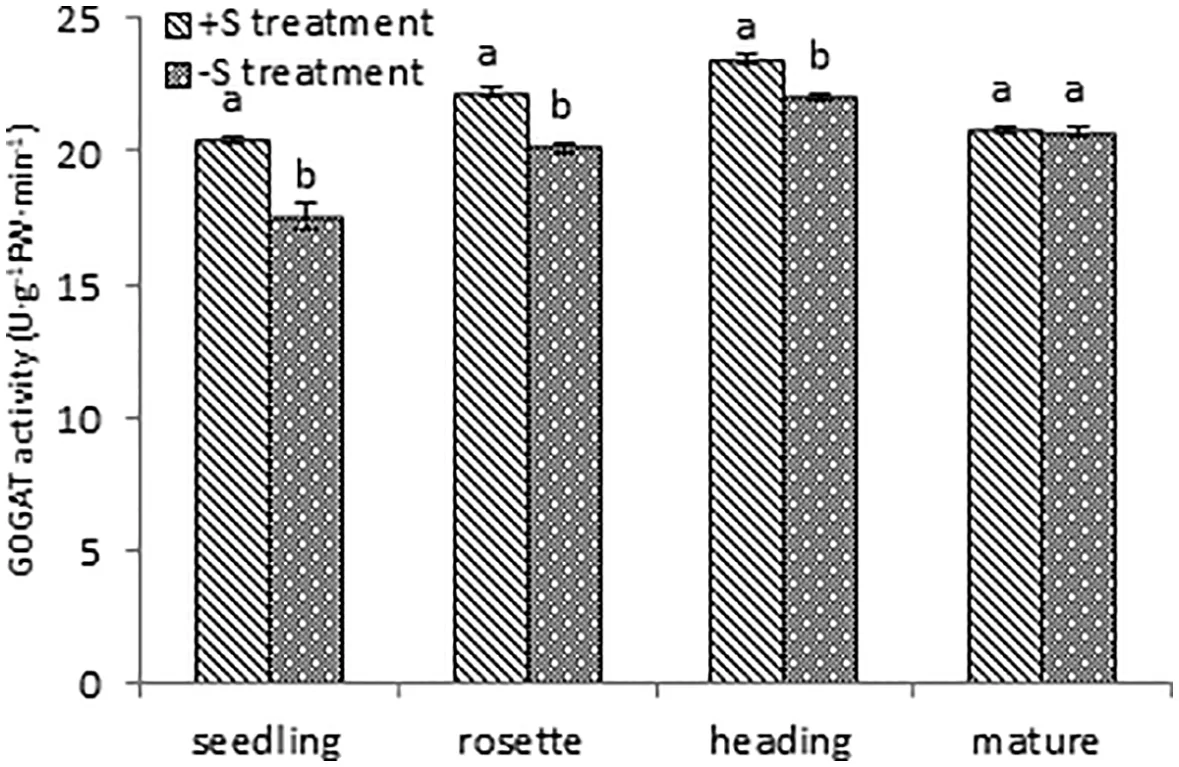
Effects of +S treatment and -S treatment on GOGAT activity of cabbage during different growth stages. Note: Different lowercase letters indicate significant differences (p < 0.05) among sulfur treatments during the same growth.

### Effect of sulfur on the expression of genes regulated by sulfur metabolism enzymes

The relative expression levels of genes related to sulfur metabolism enzymes were detected by qRT-PCR. Fig 25 shows that the expression levels of ATP sulfurylase, APS reductase, nitrite and serine acetyltransferase genes were up-regulated during the entire growth period, which is consistent with the results that the activities of APR, ATPS, SIR and SAT were significantly increased in the previous article, and the APR, ATPS, SIR and SAT genes showed a trend of first increasing and then decreasing during the entire growth period, which is consistent with the previous article. The down-regulation of O-acetylserine thiol lyase (OASTL) gene expression was contrary to the up-regulation of OASTL activity observed in the previous study, suggesting that OASTL gene plays a side regulatory role in sulfur metabolism.

**Fig 25:**
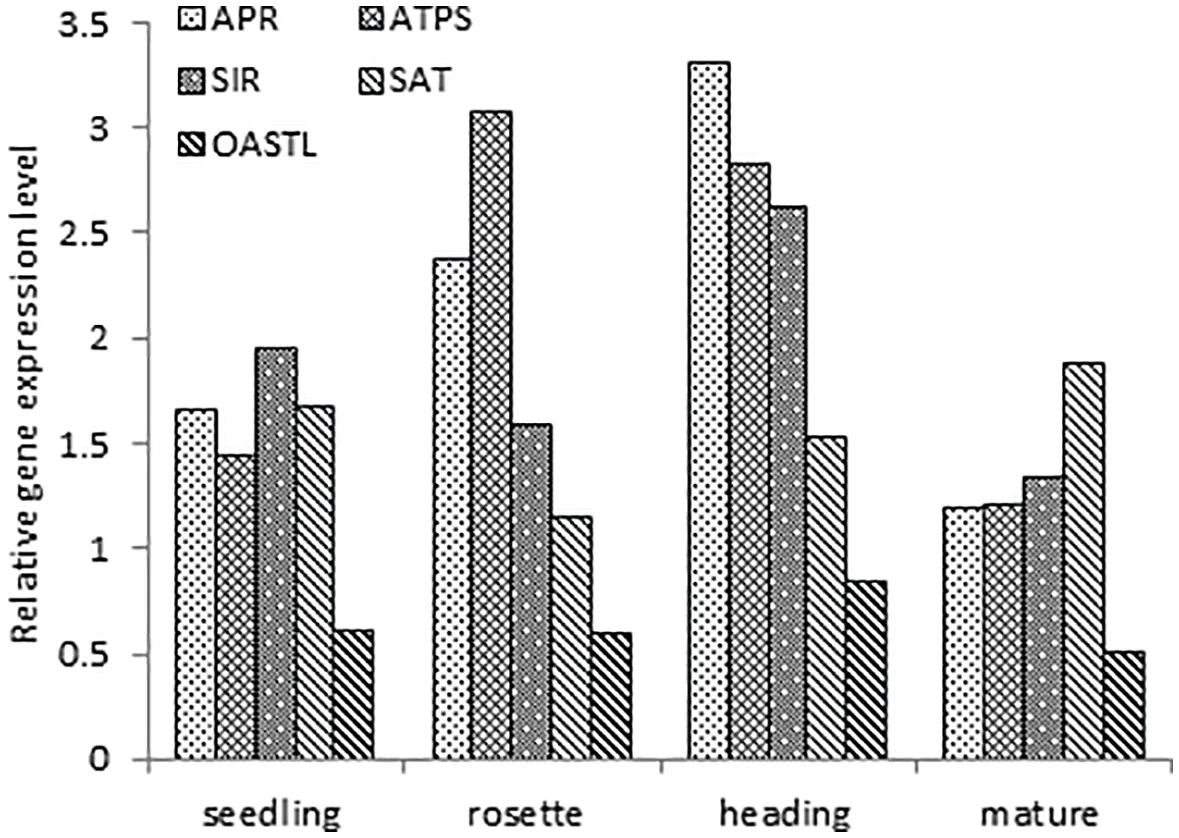
Effects of +S treatment and -S treatment gene sregulating sulfur metabolism enzymes of cabbage during different growth stages. Note: Different lowercase letters indicate significant differences (p < 0.05) among sulfur treatments during the same growth stage.

## Discussion

Sulfur is an essential nutrient for plant growth and development, and its deficiency inhibits the normal growth of plants. At the same time, sulfur deficiency also inhibits protein synthesis, activates the antioxidant system, and affects the normal growth of plants [29]. Amorim T.L et al found that under salt stress, the photosynthesis and growth of grapes were significantly inhibited [5,30]. Honsel A found that the effect of sulfur deficiency in poplars affected the growth of poplars and promoted the expression of stress resistance genes [31]. Gunes A study found that changes in physiological parameters under salt stress affected maize growth [32]. These findings are consistent with the results of the present experiment, in which plant height, stem diameter and other physiological indicators of cabbage under -S treatment were lower than those under +S treatment in the entire growth period.. The possible reasons are as follows. First, the efficiency of sulfur uptake, assimilation, and redistribution in crops is primarily determined by the expression profile of SULTR family members, which directly affects root affinity for sulfate and its transport capacity to the above-ground parts [33]. Second, adequate sulfur supply (+S) significantly enhances the activities of sulfur-assimilating enzymes such as ATP sulfurylase, APS reductase, and sulfite reductase in cabbage, while simultaneously promoting the activities of glutamine synthetase and glutamate synthase. This coordinates the metabolic flux of sulfur assimilation with nitrogen assimilation, providing sufficient carbon skeletons and reducing power for the synthesis of proteins and sulfur-containing amino acids. This metabolic synergy is ultimately reflected in the significant improvement plant height, stem diameter, and photosynthetic parameters in cabbage [34]. In addition, plant height and stem diameter under +S treatment were 34.3% and 55.9% higher than those of under -S treatment at the seedling stage, and higher than those in rosette, head, and mature stages, indicating that S had the greatest effect on the growth index of cabbage at the seedling stage. This may be due to the fact that seedling stage is the fastest growing period of cabbage and its sensitivity to stress is the highest. Furthermore, during the middle and late growth stages, cabbage leaves are larger and can absorb H_2_S from the air to alleviate the impact of -S. The main reason may be that H_2_S, as an endogenous gaseous signaling molecule, regulates key transcription factors in the ABA and IAA signaling pathways through persulfidation, thereby intrinsically modulating stomatal movement, root system architecture, and tiller initiation [35]. Chlorophyll is an important substance for light absorption, transportation and distribution during photosynthesis, and its content reflects plant photosynthesis. Liang Kuijing found that exogenous sulfur treatment increased the contents of chlorophyll a, chlorophyll b and chlorophyll (a + b) by 27.15%, 20.54% and 25.36%, respectively, and increased Pn, Tr, Gs and Ci by 28.89%, 61.76%, 17.01% and 20.57%, respectively [36]. Wang Zhucheng also demonstrated that sulfur increases chlorophyll content and significantly improved the light and ability of purslane in the study of purslane [37]. Ma Hongbo found that sulfur deficiency had a significant inhibitory effect on the chlorophyll content and Photosynthetic chlorophyll fluorescencephotosynthetic parametersin different sweet potato varieties [38]. These finfings are consistent with the results of the present study. The main reason may be that the rate of cysteine synthesis depends on reducing power derived from the photosynthetic electron transport chain, and the efficiency of sulfur reduction affects light use efficiency, thereby influencing plant photosynthetic parameters. The contents of chlorophyll a, chlorophyll b, chlorophyll a + b, and carotenoids in cabbage were significantly increased under S treatment, and Pn, Tr, Gs and Ci were also significantly increased. Stomatal and non-stomatal factors are the two main factors that reduce the photosynthetic efficiency of plants. Under abiotic stress, Pn, Gs and Ci values decrease simultaneously. It is generally accepted that stomatal factor is the main reason for the decrease of net photosynthetic rate. If the change trends of Pn and Ci are opposite, it is considered that non-stomatal factor is the main factor that reduces photosynthetic rate. Combined with the results of the present experiment, it was found that stomatal factors were the main reason for the decrease of photosynthetic rate caused by sulfur deficiency. The effect of sulfur on Photosynthetic chlorophyll fluorescencephotosynthetic parameters of cabbage often further affects the appearance quality of cabbage. In this study, we found that the leaf head of cabbage treated with -S was smaller and looser than that treated with +S. The possible reason is that -S treatment leads to low content of structural substances in leaves of cabbage or -S affects the expression of control core genes in cabbage. The specific regulatory mechanism requires further investigation.

Sulfur is the fourth most abundant nutrient element in plant growth, an important component of plant proteins and sulfur-containing amino acids, and an essential element in enzyme reactions [39]. Within the antioxidant defense system, sulfur, in the form of thiol groups, acts as a redox “sensor” on the Keap1 protein. Under oxidative stress, these thiol groups are modified, prompting Keap1 to release the transcription factor Nrf_2_. Once activated, Nrf_2_ translocates to the nucleus and initiates the expression of downstream antioxidant enzymes (such as SOD, CAT, GPx, etc.), which efficiently scavenge free radicals and protect cell membranes from lipid peroxidation. This cascade ultimately results in a significant reduction of malondialdehyde (MDA), a terminal product of lipid peroxidation. Thus, in this complete signaling chain, sulfur serves as the initial trigger, Nrf_2_ as the central commander, antioxidant enzymes as the front-line executors, and MDA as a marker of ultimate damage extent—together forming a closed-loop antioxidant defense mechanism of “sulfur modification → pathway activation → enzymatic scavenging → damage reduction.” Sulfur can activate the activity of antioxidant enzymes, increase its content and eliminate the damage caused by external stress. Ma Youhua et al. found that the activities of SOD and POD in tobacco increased significantly when sulfur fertilizer was applied in the soil with selenium fertilizer [40]. The results showed that the activities of SOD, POD, CAT and other antioxidant enzymes in rice decreased under sulfur stress. Liu Zhongliang’s research on garlic showed that increasing sulfur concentration in the nutrient solution significantly increased the activities of SOD, POD and CAT, thus effectively slowing down leaves senescence [41]. In this experiment, + S treatment significantly increased antioxidant enzyme activities in cabbage, which is consistent with previous findings. Meanwhile, the activities of SOD, POD, CAT and APX increased by 40%, 93.3%, 8.5% and 17.1%, respectively, at the seedling stage, shoeing the most significant increase compared to other stages. This indicates that the antioxidant enzyme system of cabbage was involved in the regulation of sulfur deficiency stress under -S condition, and that the seedling stage was most sensitive to sulfur deficiency stress. In conclusion, the application of sulfur significantly increased the antioxidant system enzyme activities in cabbage. The main reason may be that S promotes the enzymatic reactions, and enhances the ability of cabbage to resist external stress [42].

As an important nutrient element, the increase of S application promotes the glutathione cycle system and improves the antioxidant capacity of plants, so as to resist external stress [43]. Zembala et al. showed that the antioxidant capacity of broccoli seedlings increased after selenium stress. Reduced glutathione plays an important role in scavenging oxygen free radicals, sulfur transport and storage, enzyme activity regulation, stress signal transduction, and resistance to abiotic and biotic stresses [44]. The results showed that the content of GSH in cabbage first increased and then decreased with the growth of cabbage under -S and +S treatment, which indicated that the content of GSH in cabbage was accumulated continuously with the growth of cabbage, and the content of GSH in cabbage decreased during the heading stage to prepare for reproductive growth [45]. Sun Huili found that the GSH content in pakchoi increased significantly after sulfur application in the study of the effect of sulfur on the anti-cyclic acid-glutathione cycle of pakchoi leaves under cadmium stress. This finding is consistent with our results, in which +S treatment significantly increased the GSH content at different growth stages [46]. In this study, the change trend of GSSH content was consistent with that of GSH. This may be due to the increase of GSH content induced by +S stress, which stimulated the activity of GPx enzyme and led to the accumulation of GSSG in cabbage. As shown in Figs 14 and 15, GSH, as an important product of primary sulfur metabolism, provides reduced sulfur for GLS during its synthesis, and the increase of its content is of great significance to the synthesis of glucosinolates. Previous studies have shown that glutathione promotes glucosinolate synthesis in plants, which is consistent with the results of the present study. The possible reason are as follows: 1. Changes in the GSH/GSSG ratio may serve as a metabolic signal involved in regulating the partitioning of sulfur among different sulfur-containing compounds. 2. Sulfur deficiency conditions lead to the upregulation of miR395 expression, and this microRNA regulates sulfur supply by targeting and inhibiting the expression of ATPS genes, thereby altering the flux of the sulfur assimilation pathway. During the growth of cabbage, it needs to undergo the process of sulfur assimilation. Different sulfur forms exist at specific stages, and different sulfur metabolic enzymes regulate the transformation of different forms of sulfur. Sulfur assimilation in plants mainly comprises three phases: activation, reduction and cysteine synthesis [47]. As shown in Figs 16–20, sulfur is first absorbed by plants in the form of sulfate ion, oxidized to sulfite ion by ATPS and APR, then reduced to sulfide ion by SiR, and finally formed cysteine under the catalysis of SAT and OASTL, and then formed glucosinolate under the action of glucosinolate-related synthase. Therefore, + S and -S treatments had certain effects on the activities of sulfur metabolic enzymes and glucosinolate synthesis in cabbage. In this study, the activities of ATPS and APR in cabbage seedlings treated with +S were 15.5% and 35% higher than those treated with -S, respectively. It was shown that +S treatment promoted the transformation of sulfate ion to sulfite ion, thus promoting the increase of ATPS and APR activities, which is consistent with the finding of Miao Yujing in the study of sulfur form on Isatis indigotica growth physiology [48]. The SiR plays a key role in that process of reduce sulfite ions into sulfide ions in the cabbage. under the + S treatment, the SiR activity is obviously higher than that of the -S treatment, which is the same as the conclusion that the SiR and APR jointly control the sulfur metabolism process by Feldman-Sali [49]. The last step of cysteine synthesis in the plant sulfur assimilation pathway is catalyzed by SAT and OASTL together. In the present study, SAT and OASTL activities of cabbage under +S and-S treatments did not show the same change rule as ATPS and APR at different growth stages, which may be due to the existence of a feedback mechanism in the process of sulfur assimilation. After sulfur application, plants assimilate sulfur into organic compounds and store it in plants as soon as possible to promote plant growth. At the same time, numerous studies have shown that S directly affects the synthesis of glucosinolates. Combined with the results of this experiment and the red line in Figs 16–20, we speculate that S regulates glucosinolate synthesis by affecting the primary and secondary sulfer metabolism.

Nitrogen is first absorbed by plants in the form of nitrate ions from vitro to the body, and is catalyzed by nitrate reductase to form nitrite ions, and then NiR reduces nitrite ions to ammonia ions, and then GS and GOGAT catalyze amino acids. Sulfur assimilation and nitrogen assimilation are coordinated and inseparable in plants, and sulfur deficiency inevitably affects the process of nitrogen assimilation. Specifically, the influence of sulfur on nitrogen-metabolizing enzymes operates through a sophisticated regulatory network involving feedback regulation by sulfur-containing amino acids (cysteine), signal transduction by the metabolic intermediate O-acetylserine (OAS), and oxidative-reductive modification of enzyme proteins. Therefore, +S and -S treatments had certain effects on nitrogen metabolism enzymes of cabbage. In this study, NR activity had no obvious trend, NiR activity decreased significantly, GOGAT and GS activity increased significantly under +S treatment. The observed increase in GS and GOGAT activity under sufficient sulfur supply can be explained by the established regulatory logic: when sulfur is adequate, cysteine synthesis increases, and its accumulation exerts feedback inhibition to downregulate nitrate reductase (NR) and glutamine synthetase (GS) activities and gene expression, thereby preventing ammonium toxicity caused by excessive nitrogen assimilation. Conversely, under sulfur deficiency, cysteine synthesis is blocked, leading to accumulation of its precursor OAS, which acts as a potent signaling molecule to activate sulfur transport gene expression while relieving the inhibition on NR and even promoting its gene transcription, driving the plant to absorb more nitrate in a “search” for sulfur—thus coordinating nitrogen-sulfur balance. Saccomani found that sulfur had no significant effect on GS activity in corn [50]. It is concluded that the effects of sulfur on crop nitrogen metabolism are influenced by factors such as species, nitrogen content, sulfur forms and other factors. Additionally, the redox status of thiol groups (-SH) on enzyme molecules such as NR is subject to regulation by cellular reducing power, directly and rapidly modulating the enzymatic activity switch, and the molybdenum cofactor, for whose synthesis sulfur is essential, is also a necessary component for NR activity. The specific mechanism needs further study. It can be seen from Figs 21–24 that nitrogen affects the synthesis of glutathione and then the synthesis of glucosinolate during the assimilation process. Meanwhile, after the formation of glutamic acid, nitrogen affects the content of amino acid, the precursor of glucosinolate synthesis, and then affects the synthesis of glucosinolate through the mutual transformation between amino acids. In this study, S promoted the glutamic acid cycle pathway, promoted the increase of glutamic acid flux, and formed more downstream products(such as glutathione, tyrosine, etc.), which serves as raw materials for glucosinolate synthesis to promote glucosinolate synthesis. Nitrogen can promote the synthesis of glucosinolates. The main reason is that, through the synergistic regulation of the nitrogen-sulfur metabolic network, sulfur and nitrogen establish a functional coupling between plant growth promotion and glucosinolate metabolic induction. It should be noted that this study employed only two sulfur levels (deficient vs. sufficient) with a single concentration (2 mmol L ⁻ ¹) representing the sufficient condition. Therefore, our results do not capture the full dose-response dynamics. Future studies incorporating a gradient of sulfur concentrations are required to determine the optimal level and to fully characterize the quantitative physiological responses to sulfur supply.

## Conclusions

The results showed that sulfur-free treatment caused serious harm to the growth of cabbage, which was directly manifested as restricted growth. Particularly, the growth index of cabbage under the -S treatment was significantly lower than that under the + S treatment. At the same time, sulfur-free treatment decreased chlorophyll content, affected chlorophyll fluorescence and restricted stomatal opening. The antioxidant system and glutathione cycle system of cabbage were inhibited under sulfur-free treatment, which affected the plant resistance to external environmental stress and the synthesis of secondary metabolites. The nitrogen and sulfur metabolism pathways were also significantly inhibited under sulfur-free treatment, affecting the synthesis of secondary metabolites containing nitrogen and sulfur in plants. This study reveales the physiological mechanism of sulfur in cabbage growth, and provides a basis for the practical application of sulfur fertilizer in future plant cultivation.

## Supporting information

S1 Dataset This contains the raw experimental data and measurement values generated in this study. (XLSX)
