# Supplementary material for: Effects of sulfur on the growth and metabolism of sulfur and nitrogen in cabbage
Source: PLoS One. 2026 Nov 11;21(11):e0359848. doi: 10.1371/journal.pone.0359848 (PMC13754114; doi:10.1371/journal.pone.0359848)
Supplement: Supplementary file 1 [file pone.0359848.s002.docx]

**Comments to the Author**
Sulfur is an essential macronutrient for plants after N, P, and K. It plays a vital role in chlorophyll formation, and photosynthetic activity. It is closely linked with nitrogen metabolism and contributes to crop yield, quality, and resistance to biotic and abiotic stresses. This study investigates the effect of sulfur on growth as well as sulfur and nitrogen metabolism in cabbage. The manuscript (MS) presents novel and interesting findings and has the potential to be published in *PLOS ONE*. However, substantial revisions are required before it can be considered for publication. First, the MS should undergo a comprehensive grammatical and language review. I strongly recommend that the authors revise the MS with the assistance of a native English speaker or a professional English editing service. In addition, numerous typographical errors should be corrected. Please see my detailed comments below.

**Abstract**
The abstract is generally adequate but contains several errors and lacks key information, including details of the experimental design, the amount of sulfur used, and clarification of abbreviations. Since sulfur plays a central role in photosynthetic efficiency, chloroplast development, and nitrogen assimilation, highlighting these functions in the abstract would strengthen its scientific significance.

Line 11: Replace *was* with *is* and *necessary* with *essential*.

Line 11: Add *required* after *element*, e.g., “nutrient element required for plant growth.”

Lines 12–13: Rephrase for improved readability.

Lines 14–15: Specify the exact amount of sulfur applied.

Line 18: replace “Nitrogen metabolism and sulfur metabolism” with “Nitrogen and Sulfur metabolism”

Line 15: Insert a space after the word *solution*.

Line 23: Add photosynthesis and fluorescence parameters, as sulfur deficiency strongly impacts photosystem efficiency.

Line 26: Replace *Dis-cover* with *discovered*.

Keywords: Provide at least five keywords that are concise and not overly lengthy.

**Introduction**
The introduction is weak and lacks sufficient background. Only limited discussion is provided regarding the importance of sulfur in plant systems. Sulfur is not only critical for growth but also for the synthesis of glucosinolates and secondary metabolites, which are especially relevant for Brassica crops like cabbage. The introduction should reflect this broader role and establish why this study is significant.

Add more recent studies related to sulfur’s impact on vegetable crop yield, stress resistance, and quality.

Emphasize sulfur’s role in protein synthesis, photosynthesis, and nitrogen assimilation.

Clearly present the research hypothesis, significance, and rationale of the study.

Describe how data were collected and which statistical design was used for analysis.

Line 38: Correct *Ab-sorpation* to *absorption*.

Line 44: Replace *demon-strated* with *demonstrated*.

Line 46: Replace *en-hancing* with *enhancing*.

**Materials and Methods**
This section requires significant improvement, as critical details are missing. For a sulfur nutrition study, the form of sulfur (sulfate, elemental sulfur, etc.) and the soil’s baseline sulfur availability are critical for interpretation. Climatic conditions also influence sulfur mobility and uptake. Provide site information, including soil physicochemical characteristics, baseline sulfur levels, and climatic conditions. Clearly describe sulfur treatment methods, application amounts, and procedures. Include details on basal fertilizers and other agronomic practices. Specify the growth stages at which growth parameters were measured, as sulfur demand varies across stages. Add climate data of the site during experiment. Provide references for detailed protocols. Explain how the data were collected and which statistical design was used for analysis. If possible add experimental picture or schematic diagram.

Line 72 (Section 2.1): Add complete details of the experimental site.

Lines 92–97: Indicate the growth stage at which these indices were measured.

Lines 98–121: Specify which leaves were sampled and provide references for each protocol.

Line 122: Use the term *photosynthetic capacity* consistently throughout the manuscript.

**Results**
This section is generally acceptable, but statistical reporting is insufficient. Since sulfur significantly influences nitrogen assimilation and chlorophyll biosynthesis, more emphasis on how treatments affected these physiological traits would strengthen the section.

Lines 178–180: State the statistical significance level used to compare means.

Line 186: Specify the significance level (e.g., *p* < 0.05).

**Discussion**
The discussion should be strengthened. Sulfur’s impact on cabbage growth should be explained in terms of its role in amino acid biosynthesis, protein formation, chlorophyll content, and interaction with nitrogen. Since cabbage is a Brassica crop, sulfur’s involvement in glucosinolate synthesis and potential health-promoting compounds should also be highlighted. Provide mechanistic explanations for the results instead of mainly comparing them with previous studies. Conclude with a clear summary emphasizing the agronomic and nutritional significance of sulfur in cabbage production.

**References**

Ensure all references cited in the text appear in the reference list and vice versa.

Format references according to *PLOS ONE* guidelines.

Include recent research (within the last 5–7 years) on sulfur nutrition in Brassica crops.

**Tables**

Round all numerical values to two decimal places.

Table 3-1: Revise headings to *Chlorophyll a*, *Chlorophyll b*, and *Total chlorophyll content*.

Table 3-2: Revise the title. Use standard abbreviations in headings (e.g., CO₂, Tr, Si, Pn) with units, and explain all abbreviations in the table footnote.

**Figures**

Place figure numbering on the left side of each figure.

Figure 3-3: Improve image quality for better clarity.
