# Supplementary material for: Effects of sulfur on the growth and metabolism of sulfur and nitrogen in cabbage
Source: PLoS One. 2026 Nov 11;21(11):e0359848. doi: 10.1371/journal.pone.0359848 (PMC13754114; doi:10.1371/journal.pone.0359848)
Supplement: Supplementary file 2 [file pone.0359848.s003.docx]

PONE-D-25-34117 Revision Letter

**Journal Name**: PLOS ONE

**Manuscript Number**: PONE-D-25-34117

**Manuscript Name**: Effects of sulfur on the growth and metabolism of sulfur and nitrogen in cabbage

**Initial Date Submitted**: Jul 10 2025

**Dear Editor:**

We greatly appreciate the time and effort you and the reviewers have dedicated to reviewing our manuscript entitled " Effects of sulfur on the growth and metabolism of sulfur and nitrogen in cabbage ". We have carefully considered all the comments and suggestions, and have revised our manuscript accordingly.

Below is a point-by-point response to the reviewers' comments:

**1. Comment:**

Sulfur is an essential macronutrient for plants after N, P, and K. It plays a vital role in chlorophyll formation and photosynthetic activity. It is closely linked with nitrogen metabolism and contributes to crop yield, quality, and resistance to biotic and abiotic stresses. This study investigates the effect of sulfur on growth as well as sulfur and nitrogen metabolism in cabbage. The manuscript (MS) presents novel and interesting findings and has the potential to be published in PLOS ONE. However, substantial revisions are required before it can be considered for publication. First, the MS should undergo a comprehensive grammatical and language review. I strongly recommend that the authors revise the MS with the assistance of a native English speaker or a professional English editing service. In addition, numerous typographical errors should be corrected. Please see my detailed comments below.

**Response:**

Thank you for your insightful comment. We have revised the manuscript with the assistance of a native English speaker, and all typographical errors have been corrected.

**2. Comment:**

Abstract. The abstract is generally adequate but contains several errors and lacks key information, including details of the experimental design, the amount of sulfur used, and clarification of abbreviations. Since sulfur plays a central role in photosynthetic efficiency, chloroplast development, and nitrogen assimilation, highlighting these functions in the abstract would strengthen its scientific significance. Line 11: Replace "was" with "is" and "necessary" with "essential". Line 11: Add "required" after "element", e.g., "nutrient element required for plant growth." Lines 12–13: Rephrase for improved readability. Lines 14–15: Specify the exact amount of sulfur applied. Line 18: Replace "Nitrogen metabolism and sulfur metabolism" with "Nitrogen and Sulfur metabolism". Line 15: Insert a space after the word "solution". Line 23: Add photosynthesis and fluorescence parameters, as sulfur deficiency strongly impacts photosystem efficiency. Line 26: Replace "Dis-cover" with "discovered". Keywords: Provide at least five keywords that are concise and not overly lengthy.

**Response:**

Thank you for your insightful comment. The experimental design details and the sulfur dosage have been described in the Materials and Methods section; therefore, the sulfur dosage is not specified in the abstract. Regarding the clarification of abbreviations, "+S" represents the normal Hoagland solution used to irrigate cabbage (i.e., the sulfur‑sufficient group), and "-S" represents the sulfur‑free Hoagland solution (i.e., the sulfur‑deficient group). The descriptions of photosynthetic efficiency and nitrogen assimilation have been further refined to enhance the scientific significance of the study. The sulfur dosage has been further specified in lines 17–18. In line 29, "Nitrogen metabolism and sulfur metabolism" has been replaced with "Nitrogen and Sulfur metabolism".

**3. Comment:**

The introduction is weak and lacks sufficient background. Only limited discussion is provided regarding the importance of sulfur in plant systems. Sulfur is not only critical for growth but also for the synthesis of glucosinolates and secondary metabolites, which are especially relevant for Brassica crops like cabbage. The introduction should reflect this broader role and establish why this study is significant. Add more recent studies related to sulfur's impact on vegetable crop yield, stress resistance, and quality. Emphasize sulfur's role in protein synthesis, photosynthesis, and nitrogen assimilation. Clearly present the research hypothesis, significance, and rationale of the study. Describe how data were collected and which statistical design was used for analysis. Line 38: Correct "Ab-sorpation" to "absorption". Line 44: Replace "demon-strated" with "demonstrated". Line 46: Replace "en-hancing" with "enhancing".

**Response:**

Thank you for your insightful comment. We have carefully revised this part of the content, and the revised version is as follows: Sulfur is the fourth essential nutrient element for plants after nitrogen, phosphorus and potassium, and its absorption in plants is second only to that of nitrogen^[1]^. The sulfur absorption of cruciferous crops, especially Brassica crops, is higher than that of some grasses and more than three times the number of family crops. Sulfur exists in various forms and is abundant in the environment. It can be absorbed and utilized by plants, and plays a critical role in their growth and development. Research has demonstrated that sulfur plays essential physiological roles in plant growth, development, and stress re-sistance mechanisms^[2]^. Its metabolic regulation spans the entire plant life cycle, from seed germination to reproductive growth^[3]^. During the seed germination stage, sulfur participates in post-translational modifications of proteins by synthesizing sul-fur-containing amino acids (such as cysteine and methionine), particularly by forming disulfide bonds to stabilize the spatial conformation of proteolytic enzymes, thereby en-hancing protease activity, promoting the breakdown of storage proteins in seeds. This provides essential amino acids and nitrogen sources for rapid embryonic growth^[4]^. Dur-ing the vegetative growth stage, sulfur is a key component of the iron-sulfur (Fe-S) cluster proteins and the thioredoxin system. The former plays a central role in the electron transport chain of Photosystem I (PSI), while the latter indirectly drives chlorophyll bio-synthesis by regulating the redox status of chloroplastic enzymes^[5]^. Once sulfur becomes deficient, chlorophyll synthesis is impaired, plants exhibit leaf chlorosis, and photosyn-thetic efficiency significantly decreases, which in turn limits plant biomass accumulation. During the reproductive growth stage, sulfur is transported over long distances from ro-sette leaves to primary stems and floral organs via sulfate transporters (such as SULTR2;1). In anthers and pollen, sulfur accumulates abundantly in glutathione (GSH) to maintain the reductive potential required for pollen germination and acts as a signal-ing molecule to regulate the timing of flowering transition (bolting)^[6]^. During the late fruit development stage, sulfur serves as a precursor for sulfur-containing secondary metabo-lites. For example, in Brassicaceae plants, sulfur is incorporated into the structural back-bone of glucosinolates, whose degradation products (isothiocyanates) not only impart unique flavors to vegetables but also directly determine fruit quality characteristics^[7]^. Sulfur serves as a critical structural component of key plant enzymes and proteins, as well as a core element for chlorophyll functionality^[8]^. Its physiological roles are multifac-eted, directly promoting plant protein synthesis and enhancing photosynthetic efficiency, thereby increasing crop yield and improving nutritional quality^[9-11]^. Extensive studies have investigated sulfur's effects on staple crops such as maize^[12]^, wheat^[13]^, soybeans^[14]^, and peanuts^[15]^, with broader implications for agricultural productivity^[16]^.

Recent research has expanded to vegetable crops, revealing synergistic interactions be-tween sulfur and nitrogen assimilation pathways. These elements exhibit interdependent metabolic regulation, jointly influencing protein synthesis and enzyme activity^[17]^. In cru-ciferous crops, sulfur limitation disrupts the production of proteins and secondary me-tabolites, while optimal sulfur supplementation enhances nitrogen utilization and yield, as also demonstrated in wheat systems^[18]^. Studies in Chinese cabbage (Brassica rapa) have identified 19 BrSULTR members that are expressed in respond to salt stress and low-temperature stress^[19]^. Hooi et al. found that an anti-ESP antibody reacted with both the 39 and 35 kDa ESP forms in Brassica napus and strongly with a polypeptide corre-sponding to the 35 kDa ESP form in Crambe abyssinica^[20]^. Ma Xiaoli et al. discovered that H₂S selectively S-sulfhydrates the flowering repressor BraFLCs in Chinese cabbage^[21]^. Despite these advances, significant knowledge gaps persist regarding sulfur's influence on leafy vegetables, particularly in understanding growth dynamics and dry matter ac-cumulation, the regulatory mechanisms of the glutathione cycle, sulfur-nitrogen meta-bolic interactions, and stress adaptation responses. Underscoring warrants further ex-ploration to fully elucidate sulfur's role in leafy vegetable physiology and productivity.

In this study, 'Zhonggan 11' was used as the experimental material. By observing and measuring the effects of sulfur-sufficient (+S) and sulfur-deficient (-S) conditions on plant growth parameters, dry matter allocation, photosynthetic chlorophyll fluorescence, anti-oxidant enzyme system, glutathione cycle dynamics, nitrogen and sulfur assimilation enzymes, and key regulatory genes in the sulfur assimilation pathway, this study pro-vides fundamental insights into sulfur-mediated regulation of crop growth and estab-lishes a theoretical framework for research on the glucosinolate biosynthesis pathway in Brassicaceae crops.

**4. Comment:**

This section requires significant improvement, as critical details are missing. For a sulfur nutrition study, the form of sulfur (sulfate, elemental sulfur, etc.) and the soil's baseline sulfur availability are critical for interpretation. Climatic conditions also influence sulfur mobility and uptake. Provide site information, including soil physicochemical characteristics, baseline sulfur levels, and climatic conditions. Clearly describe sulfur treatment methods, application amounts, and procedures. Include details on basal fertilizers and other agronomic practices. Specify the growth stages at which growth parameters were measured, as sulfur demand varies across stages. Add climate data of the site during the experiment. Provide references for detailed protocols. Explain how the data were collected and which statistical design was used for analysis. If possible, add an experimental picture or schematic diagram. Line 72 (Section 2.1): Add complete details of the experimental site. Lines 92–97: Indicate the growth stage at which these indices were measured. Lines 98–121: Specify which leaves were sampled and provide references for each protocol. Line 122: Use the term "photosynthetic capacity" consistently throughout the manuscript.

**Response:**

Thank you for your insightful comment. We have revised the Materials and Methods section accordingly. The experiment was conducted in a modern intelligent greenhouse with a temperature range of 25–30 °C during the day and 16–20 °C at night. The coordinates of the experimental site are N36°05′39.86″, E103°42′31.09″. In this experiment, we used nutrient pot cultivation with a mixture of vermiculite and perlite in a 3:1 ratio, ensuring no exogenous sulfur was introduced during the experimental period. Before transplanting, all seedlings were treated with 2 mmol MgSO₄·7H₂O. After the start of the experiment, the +S treatment received 2 mmol MgSO₄·7H₂O, while the –S treatment received 2 mmol L⁻¹ MgCl₂. No base fertilizers or organic fertilizers were applied throughout the experiment; only nutrient solutions were regularly irrigated at different growth stages. Plant sampling was carried out at the seedling, rosette, expansion, and maturity stages. The corresponding references have been added to the manuscript. In lines 106–109, we have described the conditions of the experimental site. In line 119, we have stated that sampling and measurement were carried out at the seedling, rosette, heading, and mature stages. In line 120, we have described the leaf sampled: the third leaf of cabbage is a functional leaf; therefore, we selected the third leaf for measurement. We have replaced all instances of 'photosynthetic capacity' with 'photosynthetic parameters' throughout the entire manuscript. The revisions have been made according to the expert's comments, and the specific revised content is as follows: we have further enriched the introduction section and added more recent studies on the effects of sulfur on vegetable crop yield, stress resistance, and quality.

**5. Comment:**

This section is generally acceptable, but statistical reporting is insufficient. Since sulfur significantly influences nitrogen assimilation and chlorophyll biosynthesis, more emphasis on how treatments affected these physiological traits would strengthen the section. Lines 178–180: State the statistical significance level used to compare means. Line 186: Specify the significance level (e.g., p < 0.05).

**Response:**

Thank you for your insightful comment. We have added the significance level for comparing means (p < 0.05) in the manuscript.

**6. Comment:**

The discussion should be strengthened. Sulfur's impact on cabbage growth should be explained in terms of its role in amino acid biosynthesis, protein formation, chlorophyll content, and interaction with nitrogen. Since cabbage is a Brassica crop, sulfur's involvement in glucosinolate synthesis and potential health‑promoting compounds should also be highlighted. Provide mechanistic explanations for the results instead of mainly comparing them with previous studies. Conclude with a clear summary emphasizing the agronomic and nutritional significance of sulfur in cabbage production.

**Response:**

Thank you for your insightful comment. In response to the comments, we have strengthened the Discussion section. The effects of sulfur on cabbage growth are now explained from the perspectives of amino acid biosynthesis, protein formation, chlorophyll content, and sulfur–nitrogen interactions. We have also discussed the role of sulfur in glucosinolate synthesis and its contribution to potential health‑promoting compounds. The mechanistic aspects of the results are further clarified. As a result, the agronomic and nutritional significance of sulfur in cabbage production has been more prominently highlighted.

**7. Comment:**

Ensure all references cited in the text appear in the reference list and vice versa. Format references according to PLOS ONE guidelines. Include recent research (within the last 5–7 years) on sulfur nutrition in Brassica crops.

**Response:**

Thank you for your insightful comment. We have carefully verified all references in the manuscript. All references are now correctly listed in the reference section, and every entry has been revised to comply with the journal's formatting guidelines. Additionally, we have updated the references to include recent advances, with most citations drawn from studies published in the past five years.

**8. Comment:**

Round all numerical values to two decimal places. Table 3-1: Revise headings to Chlorophyll a, Chlorophyll b, and Total chlorophyll content. Table 3-2: Revise the title. Use standard abbreviations in headings (e.g., CO₂, Tr, Si, Pn) with units, and explain all abbreviations in the table footnote.

**Response:**

Thank you for your insightful comment. We have carefully addressed all of the reviewer's comments on the tables and made the necessary revisions.

**9. Comment:**

Place figure numbering on the left side of each figure. Figure 3-3: Improve image quality for better clarity.

**Response:**

Thank you for your insightful comment. We have carefully addressed all of the reviewer's comments on the figures and made the necessary revisions. The quality of Figure 3.3 is already at its highest. We have tried many approaches but still cannot effectively improve the image quality.
