# Supplementary material for: Effects of sulfur on the growth and metabolism of sulfur and nitrogen in cabbage
Source: PLoS One. 2026 Nov 11;21(11):e0359848. doi: 10.1371/journal.pone.0359848 (PMC13754114; doi:10.1371/journal.pone.0359848)
Supplement: Supplementary file 3 [file pone.0359848.s004.docx]

This manuscript addresses an important and relevant topic and provides valuable insights into the subject area. The study has potential merit, particularly in terms of its research objectives and contribution to the existing literature. However, several aspects require further clarification and improvement before the manuscript can be considered for publication.

The introduction provides a reasonable background and highlights the importance of the research question; however, the authors should strengthen the justification for the study by providing a clearer explanation of the knowledge gap and the specific contribution of this work. The methodology section requires additional details to ensure that the study design, data collection procedures, and analytical approaches are sufficiently transparent and reproducible. Any limitations related to the study design, sample size, data quality, or analytical methods should also be acknowledged and discussed appropriately.

The results are generally presented in a logical manner, but some findings require deeper interpretation and stronger linkage to the research objectives. The discussion would benefit from a more balanced evaluation of the findings in relation to previous studies, including explanations for similarities or differences with existing evidence. The conclusions should be revised to ensure that they are fully supported by the data and do not extend beyond the scope of the study.

The manuscript would also benefit from improvements in clarity, organization, and language. Figures, tables, and references should be carefully reviewed to ensure accuracy and consistency. The authors should address any ethical considerations related to the research, including appropriate approval, informed consent where applicable, and transparency regarding potential conflicts of interest. I did not identify concerns regarding duplicate publication or research misconduct; however, the authors should confirm that the work is original and has not been submitted or published elsewhere.

Overall, this is a potentially valuable study, but substantial revisions are needed to improve the methodological clarity, strengthen the interpretation of findings, and enhance the overall quality of the manuscript. Addressing these issues would improve the rigor and contribution of the work.

**Major comments**

1. **English language and grammar**

Although the language has improved, the manuscript still contains numerous grammatical errors, awkward sentence structures, typographical mistakes, and inconsistent terminology. Examples include "Sulffur," "gas excage parameters," "Photosynthetic chlorophyll fluorescencephotosynthetic parameters," inconsistent spacing around "+S" and "-S," and incorrect article usage throughout the manuscript. A thorough professional English editing is still recommended.

2. **Experimental design**

The sulfur treatment is now better described, but the manuscript still lacks sufficient justification for selecting only one sulfur concentration (2 mmol L⁻¹). The authors should explain why this concentration was chosen and discuss the limitation of using only two sulfur levels (deficient vs. sufficient) rather than a dose-response design.

3. **Statistical analysis**

The statistical analysis remains confusing. The manuscript states that both Tukey's test and Duncan's multiple range test were used. The authors should clarify which multiple comparison test was actually applied and use only one method consistently throughout the manuscript.

4. **Interpretation of results**

Several conclusions remain stronger than the presented evidence. Statements such as sulfur acting as a "central hub" regulating metabolic networks or establishing a "theoretical framework" are overstated because the study mainly presents physiological and biochemical measurements rather than mechanistic or molecular evidence. These conclusions should be moderated.

5. **Gene expression analysis**

The qRT-PCR results are briefly presented, but important methodological details remain limited. Information regarding primer validation, amplification efficiency, reference gene stability, and biological replication should be described more clearly.

6. **Discussion**

The discussion has improved considerably, but some sections remain descriptive. More emphasis should be placed on explaining the physiological mechanisms underlying sulfur-mediated regulation of nitrogen metabolism and antioxidant responses rather than simply comparing findings with previous reports.

**Minor comments**

- Carefully check all statistical significance statements. Several figure legends still report "p < 0.5" instead of "p < 0.05."
- Verify consistency in terminology (photosynthetic parameters, chlorophyll fluorescence parameters, sulfur metabolism, nitrogen metabolism).
- Ensure all abbreviations are defined upon first appearance.
- Several tables require formatting improvement, including consistent units and spacing.
- Figure legends should be edited for clarity and grammatical correctness.
- Some references cited in the discussion should be checked to ensure they directly support the corresponding statements.
- The manuscript would benefit from careful proofreading to remove remaining formatting artifacts introduced during revision.

**Publication ethics**

I have no apparent concerns regarding research ethics, publication ethics, or duplicate publication based on the information provided.

**Recommendation**

The manuscript has improved substantially after revision and contains valuable findings. However, the remaining language, statistical clarification, and interpretation issues should be addressed before the manuscript is accepted for publication. Therefore, I recommend **Minor Revision**.
