# Supplementary material for: Effects of sulfur on the growth and metabolism of sulfur and nitrogen in cabbage
Source: PLoS One. 2026 Nov 11;21(11):e0359848. doi: 10.1371/journal.pone.0359848 (PMC13754114; doi:10.1371/journal.pone.0359848)
Supplement: Supplementary file 4 [file pone.0359848.s005.docx]

PONE-D-25-34117 Revision Letter

**Journal Name**: PLOS ONE

**Manuscript Number**: PONE-D-25-34117

**Manuscript Name**: Effects of sulfur on the growth and metabolism of sulfur and nitrogen in cabbage

**Initial Date Submitted**: Jul 10 2025

**Dear Editor:**

We greatly appreciate the time and effort you and the reviewers have dedicated to reviewing our manuscript entitled " Effects of sulfur on the growth and metabolism of sulfur and nitrogen in cabbage ". We have carefully considered all the comments and suggestions, and have revised our manuscript accordingly.

Below is a point-by-point response to the reviewers' comments:

**1.English language and grammar**

Although the language has improved, the manuscript still contains numerous grammatical errors, awkward sentence structures, typographical mistakes, and inconsistent terminology. Examples include "Sulffur," "gas excage parameters," "Photosynthetic chlorophyll fluorescencephotosynthetic parameters," inconsistent spacing around "+S" and "-S," and incorrect article usage throughout the manuscript. A thorough professional English editing is still recommended.

**Response:**

Thank you for pointing out the remaining language issues. In response to your suggestion, we have engaged a professional scientific editing service to thoroughly revise the entire manuscript. Beyond correcting the grammatical errors, awkward structures, and typographical mistakes you noted (including "Sulffur," "gas excage parameters," and inconsistent terminology), the editor also standardized the format of our treatment labels (+S/-S) and refined the article usage throughout. A copy of the editing certificate has been uploaded as a supplementary file. We are confident that the language quality is now significantly improved.

**2.Experimental design**

The sulfur treatment is now better described, but the manuscript still lacks sufficient justification for selecting only one sulfur concentration (2 mmol L⁻¹). The authors should explain why this concentration was chosen and discuss the limitation of using only two sulfur levels (deficient vs. sufficient) rather than a dose-response design.

**Response:**

Thank you for your insightful comment. We appreciate the reviewer’s concern regarding the single sulfur concentration. We would like to clarify that the primary objective of our study was to elucidate the physiological and molecular mechanisms underlying sulfur deficiency stress, rather than to determine the optimal sulfur requirement for cabbage. Therefore, we adopted a binary experimental design (deficient vs. sufficient) to represent two clearly contrasting nutritional statuses, which is a standard approach in stress physiology studies.

The concentration of 2 mmol L⁻¹ corresponds to the standard sulfur supply in the widely used Hoagland nutrient solution formulation. This concentration has been consistently employed in our preliminary cultivation practices and is recognized as a sufficient and non-toxic sulfur level that supports normal growth for a broad range of plant species. Therefore, we selected this concentration as the "sufficient" treatment in our experimental design. We acknowledge that a dose-response design would provide additional valuable information, but our current experimental framework was specifically tailored to compare the "presence vs. absence" of adequate sulfur.

In response to your suggestion, we have revised the Materials and Methods section (The line:111-116) to more clearly justify the selection of 2 mmol L⁻¹ based on prior studies, and we have added a limitation statement in the Discussion acknowledging that our conclusions are confined to these two levels and that extrapolation to other concentrations requires further investigation.

**3.Statistical analysis**

The statistical analysis remains confusing. The manuscript states that both Tukey's test and Duncan's multiple range test were used. The authors should clarify which multiple comparison test was actually applied and use only one method consistently throughout the manuscript.

**Response:**

We thank the reviewer for identifying this inconsistency. We apologize for the confusion.After re-examining our data and analytical approach, we confirm that Duncan's multiple range test was the method actually used for all post-hoc comparisons. Although both tests were inadvertently mentioned in the original submission, we have now carefully revised the entire manuscript to consistently use only Duncan's test.

**4.Interpretation of results**

Several conclusions remain stronger than the presented evidence. Statements such as sulfur acting as a "central hub" regulating metabolic networks or establishing a "theoretical framework" are overstated because the study mainly presents physiological and biochemical measurements rather than mechanistic or molecular evidence. These conclusions should be moderated.

**Response:**

We greatly appreciate the reviewer's thoughtful comments. Accordingly, we have carefully revised both the Introduction and the Conclusion sections to ensure that all descriptions remain strictly focused within the scope of our study. Content that was deemed beyond the scope of this work has been either removed or appropriately rephrased.

**5.Gene expression analysis**

The qRT-PCR results are briefly presented, but important methodological details remain limited. Information regarding primer validation, amplification efficiency, reference gene stability, and biological replication should be described more clearly.

**Response:**

We sincerely thank you for your careful review and valuable comments on our manuscript. We have thoroughly considered your suggestions and have revised the manuscript accordingly. Below we provide our point-by-point responses to your concerns.

We fully agree with the reviewer’s assessment that methodological details are critical for the reproducibility of qRT-PCR results. Accordingly, we have significantly expanded the description of our qRT-PCR methodology in the Materials and Methods section.

Specifically, we have now added the following details:

Primer design and validation: All primers were designed using Primer Premier 5.0 based on the cDNA sequences deposited in GenBank.

Amplification efficiency: All primers used in this study exhibited amplification efficiencies ranging from 92% to 105% (R² > 0.99).

Replication: All qRT-PCR reactions were performed in triplicate technical repeats, and three independent biological replicates were conducted for each treatment.

Quantification method: The relative expression levels of target genes were calculated using the 2^⁻ΔΔCt^ method after normalization to the selected reference gene.

We believe these additions substantially improve the methodological transparency and reproducibility of our study. We are grateful for the reviewer’s constructive suggestion, which has helped us strengthen our manuscript.

**6.Discussion**

The discussion has improved considerably, but some sections remain descriptive. More emphasis should be placed on explaining the physiological mechanisms underlying sulfur-mediated regulation of nitrogen metabolism and antioxidant responses rather than simply comparing findings with previous reports.

**Response:**

We sincerely thank the reviewer for this insightful and constructive comment. We fully agree that a deeper mechanistic explanation is far more valuable than simple data comparison. Accordingly, we have substantially revised the Discussion section with the following improvements:

In the nitrogen metabolism subsection, we have elucidated the mechanistic influence of sulfur on nitrogen-metabolizing enzymes. Sulfur operates through a sophisticated regulatory network involving three interconnected mechanisms: feedback regulation by sulfur-containing amino acids (cysteine), signal transduction by the metabolic intermediate O-acetylserine (OAS), and oxidative-reductive modification of enzyme proteins. When sulfur supply is adequate, cysteine synthesis increases, and its accumulation exerts feedback inhibition to downregulate the activities and gene expression of key nitrogen-metabolizing enzymes such as nitrate reductase (NR) and glutamine synthetase (GS), thereby preventing ammonium toxicity caused by excessive nitrogen assimilation. Conversely, under sulfur deficiency, cysteine synthesis is blocked, leading to accumulation of its precursor OAS, which acts as a potent signaling molecule to activate sulfur transport gene expression while simultaneously relieving the inhibition on NR and even promoting its gene transcription, driving the plant to absorb more nitrate in a "search" for sulfur—thus coordinating nitrogen-sulfur balance. In addition, the redox status of thiol groups (-SH) on enzyme molecules such as NR is subject to regulation by cellular reducing power, directly and rapidly modulating the enzymatic activity switch, and the molybdenum cofactor, for whose synthesis sulfur is essential, is also a necessary component for NR activity.

In the antioxidant response subsection, we have elucidated that sulfur, in the form of thiol groups, acts as a redox "sensor" on the Keap1 protein. When the organism is subjected to oxidative stress, these thiol groups are modified, prompting Keap1 to release the transcription factor Nrf2. Once activated, Nrf2 translocates to the nucleus and initiates the expression of downstream antioxidant enzymes (such as SOD, CAT, GPx, etc.). These enzymes efficiently scavenge free radicals to protect cell membranes from lipid peroxidation attack, and the ultimate effect is directly reflected as a significant reduction in the production of malondialdehyde (MDA), a terminal product of lipid peroxidation. Therefore, within this complete signaling chain, sulfur serves as the initial trigger, Nrf2 as the central commander, antioxidant enzymes as the front-line executors, and MDA as a marker of ultimate damage extent—together forming a closed-loop antioxidant defense mechanism of "sulfur modification → pathway activation → enzymatic scavenging → damage reduction."

All revisions have been using track changes for your easy reference. We hope these modifications adequately address your concern.

**7.Minor comments**

Carefully check all statistical significance statements. Several figure legends still report "p < 0.5" instead of "p < 0.05."

Verify consistency in terminology (photosynthetic parameters, chlorophyll fluorescence parameters, sulfur metabolism, nitrogen metabolism).

Ensure all abbreviations are defined upon first appearance.

Several tables require formatting improvement, including consistent units and spacing.

Figure legends should be edited for clarity and grammatical correctness.

Some references cited in the discussion should be checked to ensure they directly support the corresponding statements.

The manuscript would benefit from careful proofreading to remove remaining formatting artifacts introduced during revision.

**Response:**

We sincerely thank the reviewer for this thorough and meticulous review. These technical and editorial points are extremely valuable for improving the overall quality and clarity of our manuscript. We have carefully addressed each issue as detailed below:

1. Statistical significance statements. We have meticulously checked all significance indicators throughout the manuscript. All instances of "p < 0.5" have been corrected to the proper threshold of "p < 0.05" in both the text and all figure legends. We have also verified that all other statistical notations (e.g., p < 0.01, p < 0.001) are correctly reported.

2. Terminology consistency. We have systematically reviewed the entire manuscript to ensure consistent usage of the following key terms: photosynthetic parameters (e.g., Pn, Gs, Ci, Tr), chlorophyll fluorescence parameters (e.g., Fv/Fm, ΦPSII, qP, NPQ), sulfur metabolism (e.g., sulfate uptake, cysteine biosynthesis, glutathione pool), and nitrogen metabolism (e.g., nitrate reductase, glutamine synthetase, nitrogen assimilation). All terms are now used uniformly throughout the text, tables, and figures.

3. Abbreviation definitions. We have verified that every abbreviation is defined upon its first appearance in the abstract, main text, and each figure legend. A comprehensive list of abbreviations has also been added (or updated) for easy reference.

4. Table formatting. All tables have been reformatted to improve readability. We have ensured consistent units are provided for all parameters and that appropriate spacing has been applied between columns and rows. Units are now uniformly placed in the column headers rather than repeated in each cell.

5. Figure legends. We have carefully edited all figure legends for clarity and grammatical correctness. Redundant or ambiguous descriptions have been revised to ensure each legend is self-explanatory and accurately reflects the corresponding figure content.

6. Reference verification. We have re‑examined all references cited in the Discussion section to confirm that each one directly supports the corresponding statement. Where necessary, we have replaced or supplemented references with more relevant citations.

7. Proofreading. The entire manuscript has undergone careful proofreading to remove formatting artifacts and typographical errors introduced during the revision process. We have also checked line spacing, font consistency, and reference formatting.

All revisions have been using track changes for your easy reference. We hope these improvements meet your expectations.
