# Supplementary material for: Effects of sulfur on the growth and metabolism of sulfur and nitrogen in cabbage
Source: PLoS One. 2026 Nov 11;21(11):e0359848. doi: 10.1371/journal.pone.0359848 (PMC13754114; doi:10.1371/journal.pone.0359848)
Supplement: Peer review history

# Peer review: Decision Letter 0

**DOI:** 10.1371/journal.pone.0359848.r001

**Authors:** Trung Quang Nguyen

12 Feb 2026

Thank you for submitting your manuscript to PLOS ONE. After careful consideration, we feel that it has merit but does not fully meet PLOS ONE’s publication criteria as it currently stands. Therefore, we invite you to submit a revised version of the manuscript that addresses the points raised during the review process.

Please submit your revised manuscript by Mar 29 2026 11:59PM. If you will need more time than this to complete your revisions, please reply to this message or contact the journal office at plosone@plos.org. When you're ready to submit your revision, log on to https://www.editorialmanager.com/pone/ and select the 'Submissions Needing Revision' folder to locate your manuscript file.

- A letter that responds to each point raised by the academic editor and reviewer(s). You should upload this letter as a separate file labeled 'Response to Reviewers'.
- A marked-up copy of your manuscript that highlights changes made to the original version. You should upload this as a separate file labeled 'Revised Manuscript with Track Changes'.
- An unmarked version of your revised paper without tracked changes. You should upload this as a separate file labeled 'Manuscript'.

If you would like to make changes to your financial disclosure, please include your updated statement in your cover letter. Guidelines for resubmitting your figure files are available below the reviewer comments at the end of this letter.

If applicable, we recommend that you deposit your laboratory protocols in protocols.io to enhance the reproducibility of your results. Protocols.io assigns your protocol its own identifier (DOI) so that it can be cited independently in the future. For instructions see: https://journals.plos.org/plosone/s/submission-guidelines#loc-laboratory-protocols. Additionally, PLOS ONE offers an option for publishing peer-reviewed Lab Protocol articles, which describe protocols hosted on protocols.io. Read more information on sharing protocols at https://plos.org/protocols?utm_medium=editorial-email&utm_source=authorletters&utm_campaign=protocols.

We look forward to receiving your revised manuscript.

Kind regards,

Trung Quang Nguyen

Academic Editor

PLOS One

Journal Requirements:

When submitting your revision, we need you to address these additional requirements.

1. Please ensure that your manuscript meets PLOS ONE’s style requirements, including those for file naming. The PLOS ONE style templates can be found at

https://journals.plos.org/plosone/s/file?id=wjVg/PLOSOne_formatting_sample_main_body.pdf and

https://journals.plos.org/plosone/s/file?id=ba62/PLOSOne_formatting_sample_title_authors_affiliations.pdf

2. We note that your Data Availability Statement is currently as follows: All relevant data are within the manuscript and its Supporting Information files.

Please confirm at this time whether or not your submission contains all raw data required to replicate the results of your study. Authors must share the “minimal data set” for their submission. PLOS defines the minimal data set to consist of the data required to replicate all study findings reported in the article, as well as related metadata and methods (https://journals.plos.org/plosone/s/data-availability#loc-minimal-data-set-definition).

For example, authors should submit the following data:

- The values behind the means, standard deviations and other measures reported;

- The values used to build graphs;

- The points extracted from images for analysis.

Authors do not need to submit their entire data set if only a portion of the data was used in the reported study.

If your submission does not contain these data, please either upload them as Supporting Information files or deposit them to a stable, public repository and provide us with the relevant URLs, DOIs, or accession numbers. For a list of recommended repositories, please see https://journals.plos.org/plosone/s/recommended-repositories.

If there are ethical or legal restrictions on sharing a de-identified data set, please explain them in detail (e.g., data contain potentially sensitive information, data are owned by a third-party organization, etc.) and who has imposed them (e.g., an ethics committee). Please also provide contact information for a data access committee, ethics committee, or other institutional body to which data requests may be sent. If data are owned by a third party, please indicate how others may request data access.

3. When completing the data availability statement of the submission form, you indicated that you will make your data available on acceptance. We strongly recommend all authors decide on a data sharing plan before acceptance, as the process can be lengthy and hold up publication timelines. Please note that, though access restrictions are acceptable now, your entire data will need to be made freely accessible if your manuscript is accepted for publication. This policy applies to all data except where public deposition would breach compliance with the protocol approved by your research ethics board. If you are unable to adhere to our open data policy, please kindly revise your statement to explain your reasoning and we will seek the editor’s input on an exemption. Please be assured that, once you have provided your new statement, the assessment of your exemption will not hold up the peer review process.

4. Please amend the manuscript submission data (via Edit Submission) to include author YuDan Deng

5. Please amend your authorship list in your manuscript file to include author Deng Yundan

6. Please upload a copy of Figures 2-5, 2-8, and 2-9 to which you refer in your text on pages 9, 14, and 15. If the figures are no longer to be included as part of the submission please remove all reference to it within the text.

7. Please include a copy of Table 2-2 which you refer to in your text on page 7.

8. We note you have included a table to which you do not refer in the text of your manuscript. Please ensure that you refer to Tables 2-1 and 3-2 in your text; if accepted, production will need this reference to link the reader to the Tables.

9. If the reviewer comments include a recommendation to cite specific previously published works, please review and evaluate these publications to determine whether they are relevant and should be cited. There is no requirement to cite these works unless the editor has indicated otherwise.

[Note: HTML markup is below. Please do not edit.]

Reviewers' comments:

Reviewer’s Responses to Questions

**Comments to the Author**

1. Is the manuscript technically sound, and do the data support the conclusions?

Reviewer #1: Yes

Reviewer #2: Partly

**********

2. Has the statistical analysis been performed appropriately and rigorously?

Reviewer #1: Yes

Reviewer #2: No

**********

3. Have the authors made all data underlying the findings in their manuscript fully available?

The PLOS Data policy

Reviewer #1: Yes

Reviewer #2: Yes

**********

4. Is the manuscript presented in an intelligible fashion and written in standard English?

Reviewer #1: No

Reviewer #2: No

**********

Reviewer #1: Comments to the Author

Sulfur is an essential macronutrient for plants after N, P, and K. It plays a vital role in chlorophyll formation, and photosynthetic activity. It is closely linked with nitrogen metabolism and contributes to crop yield, quality, and resistance to biotic and abiotic stresses. This study investigates the effect of sulfur on growth as well as sulfur and nitrogen metabolism in cabbage. The manuscript (MS) presents novel and interesting findings and has the potential to be published in PLOS ONE. However, substantial revisions are required before it can be considered for publication. First, the MS should undergo a comprehensive grammatical and language review. I strongly recommend that the authors revise the MS with the assistance of a native English speaker or a professional English editing service. In addition, numerous typographical errors should be corrected. Please see my detailed comments below.

Abstract

The abstract is generally adequate but contains several errors and lacks key information, including details of the experimental design, the amount of sulfur used, and clarification of abbreviations. Since sulfur plays a central role in photosynthetic efficiency, chloroplast development, and nitrogen assimilation, highlighting these functions in the abstract would strengthen its scientific significance.

Line 11: Replace was with is and necessary with essential.

Line 11: Add required after element, e.g., “nutrient element required for plant growth.”

Lines 12–13: Rephrase for improved readability.

Lines 14–15: Specify the exact amount of sulfur applied.

Line 18: replace “Nitrogen metabolism and sulfur metabolism” with “Nitrogen and Sulfur metabolism”

Line 15: Insert a space after the word solution.

Line 23: Add photosynthesis and fluorescence parameters, as sulfur deficiency strongly impacts photosystem efficiency.

Line 26: Replace Dis-cover with discovered.

Keywords: Provide at least five keywords that are concise and not overly lengthy.

Introduction

The introduction is weak and lacks sufficient background. Only limited discussion is provided regarding the importance of sulfur in plant systems. Sulfur is not only critical for growth but also for the synthesis of glucosinolates and secondary metabolites, which are especially relevant for Brassica crops like cabbage. The introduction should reflect this broader role and establish why this study is significant.

Add more recent studies related to sulfur’s impact on vegetable crop yield, stress resistance, and quality.

Emphasize sulfur’s role in protein synthesis, photosynthesis, and nitrogen assimilation.

Clearly present the research hypothesis, significance, and rationale of the study.

Describe how data were collected and which statistical design was used for analysis.

Line 38: Correct Ab-sorpation to absorption.

Line 44: Replace demon-strated with demonstrated.

Line 46: Replace en-hancing with enhancing.

Materials and Methods

This section requires significant improvement, as critical details are missing. For a sulfur nutrition study, the form of sulfur (sulfate, elemental sulfur, etc.) and the soil’s baseline sulfur availability are critical for interpretation. Climatic conditions also influence sulfur mobility and uptake. Provide site information, including soil physicochemical characteristics, baseline sulfur levels, and climatic conditions. Clearly describe sulfur treatment methods, application amounts, and procedures. Include details on basal fertilizers and other agronomic practices. Specify the growth stages at which growth parameters were measured, as sulfur demand varies across stages. Add climate data of the site during experiment. Provide references for detailed protocols. Explain how the data were collected and which statistical design was used for analysis. If possible add experimental picture or schematic diagram.

Line 72 (Section 2.1): Add complete details of the experimental site.

Lines 92–97: Indicate the growth stage at which these indices were measured.

Lines 98–121: Specify which leaves were sampled and provide references for each protocol.

Line 122: Use the term photosynthetic capacity consistently throughout the manuscript.

Results

This section is generally acceptable, but statistical reporting is insufficient. Since sulfur significantly influences nitrogen assimilation and chlorophyll biosynthesis, more emphasis on how treatments affected these physiological traits would strengthen the section.

Lines 178–180: State the statistical significance level used to compare means.

Line 186: Specify the significance level (e.g., p < 0.05).

Discussion

The discussion should be strengthened. Sulfur’s impact on cabbage growth should be explained in terms of its role in amino acid biosynthesis, protein formation, chlorophyll content, and interaction with nitrogen. Since cabbage is a Brassica crop, sulfur’s involvement in glucosinolate synthesis and potential health-promoting compounds should also be highlighted. Provide mechanistic explanations for the results instead of mainly comparing them with previous studies. Conclude with a clear summary emphasizing the agronomic and nutritional significance of sulfur in cabbage production.

References

Ensure all references cited in the text appear in the reference list and vice versa.

Format references according to PLOS ONE guidelines.

Include recent research (within the last 5–7 years) on sulfur nutrition in Brassica crops.

Tables

Round all numerical values to two decimal places.

Table 3-1: Revise headings to Chlorophyll a, Chlorophyll b, and Total chlorophyll content.

Table 3-2: Revise the title. Use standard abbreviations in headings (e.g., CO₂, Tr, Si, Pn) with units, and explain all abbreviations in the table footnote.

Figures

Place figure numbering on the left side of each figure.

Figure 3-3: Improve image quality for better clarity.

Reviewer #2: Review

1. Kindly attend to some grammar adjustments, typographical errors, and spacing needed throughout the manuscript.

a. The first two sentences in the abstract were not clear

b. Some words that needed no hyphen had a hyphen in them, e.g., dis-covered line 25, fer-tilizer line 32, en-hancing line 46, seed-ling line 76………

c. Some needed spacing after a unit or after a full stop, see lines 144, 145, 150……..

2. Why did the study use ‘Zhonggan 11’? Did it affect the outcome of the study?

3. Is the information in lines 86-90 needed for this manuscript? Research manuscripts reporting large……….

4. In the antioxidant enzyme assay, experiments were performed according to the kit instructions. Kindly provide information about the kit’s manufacturer/catalog. This also applies to all cases where kits were reported to be used across the manuscript.

5. Line 133, 9.00-11.00 in the morning, suggests that the experiment was not carried out in a greenhouse. What other environmental conditions might affect the result? Also, provide information on the season, month, and other relevant details. The experiment was carried out.

6. Line 148 reads spinach leaves. Is this a mistake?

7. Some figures were cited that are missing Fig. 2-5 line 308, line 453, line 498

8. The first paragraph of the discussion suggests that stress was inferred, since its effect was not directly measured.

9. Is the statistical package used for the study appropriate to detect the level of significance? Also, why is the p-value less than 0.5 in some cases, and 0.05 in others?

10. Kindly cite authors correctly; see lines 377-378 and elsewhere.

11. Kindly attend to figure numbering; Fig 3-1……..

12. Concentration of Sulphur used in the study and why

**********

what does this mean?). If published, this will include your full peer review and any attached files.

If you choose “no”, your identity will remain anonymous but your review may still be made public.

**Do you want your identity to be public for this peer review?**  For information about this choice, including consent withdrawal, please see our Privacy Policy

Reviewer #1: No

Reviewer #2: No

**********

[NOTE: If reviewer comments were submitted as an attachment file, they will be attached to this email and accessible via the submission site. Please log into your account, locate the manuscript record, and check for the action link "View Attachments". If this link does not appear, there are no attachment files.]

To ensure your figures meet our technical requirements, please review our figure guidelines: https://journals.plos.org/plosone/s/figures

You may also use PLOS’s free figure tool, NAAS, to help you prepare publication quality figures: https://journals.plos.org/plosone/s/figures#loc-tools-for-figure-preparation.

NAAS will assess whether your figures meet our technical requirements by comparing each figure against our figure specifications.

Attachment Submitted filename: Comments to the Author (PLOS ONE).docx

# Author comment: Author response to Decision Letter 1

**DOI:** 10.1371/journal.pone.0359848.r002

11 Jun 2026

PONE-D-25-34117 Revision Letter

Journal Name: PLOS ONE

Manuscript Number: PONE-D-25-34117

Manuscript Name: Effects of sulfur on the growth and metabolism of sulfur and nitrogen in cabbage

Initial Date Submitted: Jul 10 2025

Dear Editor:

We greatly appreciate the time and effort you and the reviewers have dedicated to reviewing our manuscript entitled " Effects of sulfur on the growth and metabolism of sulfur and nitrogen in cabbage ". We have carefully considered all the comments and suggestions, and have revised our manuscript accordingly.

Below is a point-by-point response to the reviewers' comments:

1. Comment:

Sulfur is an essential macronutrient for plants after N, P, and K. It plays a vital role in chlorophyll formation and photosynthetic activity. It is closely linked with nitrogen metabolism and contributes to crop yield, quality, and resistance to biotic and abiotic stresses. This study investigates the effect of sulfur on growth as well as sulfur and nitrogen metabolism in cabbage. The manuscript (MS) presents novel and interesting findings and has the potential to be published in PLOS ONE. However, substantial revisions are required before it can be considered for publication. First, the MS should undergo a comprehensive grammatical and language review. I strongly recommend that the authors revise the MS with the assistance of a native English speaker or a professional English editing service. In addition, numerous typographical errors should be corrected. Please see my detailed comments below.

Response:

Thank you for your insightful comment. We have revised the manuscript with the assistance of a native English speaker, and all typographical errors have been corrected.

2. Comment:

Abstract. The abstract is generally adequate but contains several errors and lacks key information, including details of the experimental design, the amount of sulfur used, and clarification of abbreviations. Since sulfur plays a central role in photosynthetic efficiency, chloroplast development, and nitrogen assimilation, highlighting these functions in the abstract would strengthen its scientific significance. Line 11: Replace "was" with "is" and "necessary" with "essential". Line 11: Add "required" after "element", e.g., "nutrient element required for plant growth." Lines 12–13: Rephrase for improved readability. Lines 14–15: Specify the exact amount of sulfur applied. Line 18: Replace "Nitrogen metabolism and sulfur metabolism" with "Nitrogen and Sulfur metabolism". Line 15: Insert a space after the word "solution". Line 23: Add photosynthesis and fluorescence parameters, as sulfur deficiency strongly impacts photosystem efficiency. Line 26: Replace "Dis-cover" with "discovered". Keywords: Provide at least five keywords that are concise and not overly lengthy.

Response:

Thank you for your insightful comment. The experimental design details and the sulfur dosage have been described in the Materials and Methods section; therefore, the sulfur dosage is not specified in the abstract. Regarding the clarification of abbreviations, "+S" represents the normal Hoagland solution used to irrigate cabbage (i.e., the sulfur‑sufficient group), and "-S" represents the sulfur‑free Hoagland solution (i.e., the sulfur‑deficient group). The descriptions of photosynthetic efficiency and nitrogen assimilation have been further refined to enhance the scientific significance of the study. The sulfur dosage has been further specified in lines 17–18. In line 29, "Nitrogen metabolism and sulfur metabolism" has been replaced with "Nitrogen and Sulfur metabolism".

3. Comment:

The introduction is weak and lacks sufficient background. Only limited discussion is provided regarding the importance of sulfur in plant systems. Sulfur is not only critical for growth but also for the synthesis of glucosinolates and secondary metabolites, which are especially relevant for Brassica crops like cabbage. The introduction should reflect this broader role and establish why this study is significant. Add more recent studies related to sulfur’s impact on vegetable crop yield, stress resistance, and quality. Emphasize sulfur’s role in protein synthesis, photosynthesis, and nitrogen assimilation. Clearly present the research hypothesis, significance, and rationale of the study. Describe how data were collected and which statistical design was used for analysis. Line 38: Correct "Ab-sorpation" to "absorption". Line 44: Replace "demon-strated" with "demonstrated". Line 46: Replace "en-hancing" with "enhancing".

Response:

Thank you for your insightful comment. We have carefully revised this part of the content, and the revised version is as follows: Sulfur is the fourth essential nutrient element for plants after nitrogen, phosphorus and potassium, and its absorption in plants is second only to that of nitrogen[1]. The sulfur absorption of cruciferous crops, especially Brassica crops, is higher than that of some grasses and more than three times the number of family crops. Sulfur exists in various forms and is abundant in the environment. It can be absorbed and utilized by plants, and plays a critical role in their growth and development. Research has demonstrated that sulfur plays essential physiological roles in plant growth, development, and stress re-sistance mechanisms[2]. Its metabolic regulation spans the entire plant life cycle, from seed germination to reproductive growth[3]. During the seed germination stage, sulfur participates in post-translational modifications of proteins by synthesizing sul-fur-containing amino acids (such as cysteine and methionine), particularly by forming disulfide bonds to stabilize the spatial conformation of proteolytic enzymes, thereby en-hancing protease activity, promoting the breakdown of storage proteins in seeds. This provides essential amino acids and nitrogen sources for rapid embryonic growth[4]. Dur-ing the vegetative growth stage, sulfur is a key component of the iron-sulfur (Fe-S) cluster proteins and the thioredoxin system. The former plays a central role in the electron transport chain of Photosystem I (PSI), while the latter indirectly drives chlorophyll bio-synthesis by regulating the redox status of chloroplastic enzymes[5]. Once sulfur becomes deficient, chlorophyll synthesis is impaired, plants exhibit leaf chlorosis, and photosyn-thetic efficiency significantly decreases, which in turn limits plant biomass accumulation. During the reproductive growth stage, sulfur is transported over long distances from ro-sette leaves to primary stems and floral organs via sulfate transporters (such as SULTR2;1). In anthers and pollen, sulfur accumulates abundantly in glutathione (GSH) to maintain the reductive potential required for pollen germination and acts as a signal-ing molecule to regulate the timing of flowering transition (bolting)[6]. During the late fruit development stage, sulfur serves as a precursor for sulfur-containing secondary metabo-lites. For example, in Brassicaceae plants, sulfur is incorporated into the structural back-bone of glucosinolates, whose degradation products (isothiocyanates) not only impart unique flavors to vegetables but also directly determine fruit quality characteristics[7]. Sulfur serves as a critical structural component of key plant enzymes and proteins, as well as a core element for chlorophyll functionality[8]. Its physiological roles are multifac-eted, directly promoting plant protein synthesis and enhancing photosynthetic efficiency, thereby increasing crop yield and improving nutritional quality[9-11]. Extensive studies have investigated sulfur’s effects on staple crops such as maize[12], wheat[13], soybeans[14], and peanuts[15], with broader implications for agricultural productivity[16].

Recent research has expanded to vegetable crops, revealing synergistic interactions be-tween sulfur and nitrogen assimilation pathways. These elements exhibit interdependent metabolic regulation, jointly influencing protein synthesis and enzyme activity[17]. In cru-ciferous crops, sulfur limitation disrupts the production of proteins and secondary me-tabolites, while optimal sulfur supplementation enhances nitrogen utilization and yield, as also demonstrated in wheat systems[18]. Studies in Chinese cabbage (Brassica rapa) have identified 19 BrSULTR members that are expressed in respond to salt stress and low-temperature stress[19]. Hooi et al. found that an anti-ESP antibody reacted with both the 39 and 35 kDa ESP forms in Brassica napus and strongly with a polypeptide corre-sponding to the 35 kDa ESP form in Crambe abyssinica[20]. Ma Xiaoli et al. discovered that H₂S selectively S-sulfhydrates the flowering repressor BraFLCs in Chinese cabbage[21]. Despite these advances, significant knowledge gaps persist regarding sulfur’s influence on leafy vegetables, particularly in understanding growth dynamics and dry matter ac-cumulation, the regulatory mechanisms of the glutathione cycle, sulfur-nitrogen meta-bolic interactions, and stress adaptation responses. Underscoring warrants further ex-ploration to fully elucidate sulfur’s role in leafy vegetable physiology and productivity.

In this study, 'Zhonggan 11' was used as the experimental material. By observing and measuring the effects of sulfur-sufficient (+S) and sulfur-deficient (-S) conditions on plant growth parameters, dry matter allocation, photosynthetic chlorophyll fluorescence, anti-oxidant enzyme system, glutathione cycle dynamics, nitrogen and sulfur assimilation enzymes, and key regulatory genes in the sulfur assimilation pathway, this study pro-vides fundamental insights into sulfur-mediated regulation of crop growth and estab-lishes a theoretical framework for research on the glucosinolate biosynthesis pathway in Brassicaceae crops.

4. Comment:

This section requires significant improvement, as critical details are missing. For a sulfur nutrition study, the form of sulfur (sulfate, elemental sulfur, etc.) and the soil’s baseline sulfur availability are critical for interpretation. Climatic conditions also influence sulfur mobility and uptake. Provide site information, including soil physicochemical characteristics, baseline sulfur levels, and climatic conditions. Clearly describe sulfur treatment methods, application amounts, and procedures. Include details on basal fertilizers and other agronomic practices. Specify the growth stages at which growth parameters were measured, as sulfur demand varies across stages. Add climate data of the site during the experiment. Provide references for detailed protocols. Explain how the data were collected and which statistical design was used for analysis. If possible, add an experimental picture or schematic diagram. Line 72 (Section 2.1): Add complete details of the experimental site. Lines 92–97: Indicate the growth stage at which these indices were measured. Lines 98–121: Specify which leaves were sampled and provide references for each protocol. Line 122: Use the term "photosynthetic capacity" consistently throughout the manuscript.

Response:

Thank you for your insightful comment. We have revised the Materials and Methods section accordingly. The experiment was conducted in a modern intelligent greenhouse with a temperature range of 25–30 °C during the day and 16–20 °C at night. The coordinates of the experimental site are N36°05′39.86″, E103°42′31.09″. In this experiment, we used nutrient pot cultivation with a mixture of vermiculite and perlite in a 3:1 ratio, ensuring no exogenous sulfur was introduced during the experimental period. Before transplanting, all seedlings were treated with 2 mmol MgSO₄·7H₂O. After the start of the experiment, the +S treatment received 2 mmol MgSO₄·7H₂O, while the –S treatment received 2 mmol L⁻¹ MgCl₂. No base fertilizers or organic fertilizers were applied throughout the experiment; only nutrient solutions were regularly irrigated at different growth stages. Plant sampling was carried out at the seedling, rosette, expansion, and maturity stages. The corresponding references have been added to the manuscript. In lines 106–109, we have described the conditions of the experimental site. In line 119, we have stated that sampling and measurement were carried out at the seedling, rosette, heading, and mature stages. In line 120, we have described the leaf sampled: the third leaf of cabbage is a functional leaf; therefore, we selected the third leaf for measurement. We have replaced all instances of 'photosynthetic capacity' with 'photosynthetic parameters' throughout the entire manuscript. The revisions have been made according to the expert’s comments, and the specific revised content is as follows: we have further enriched the introduction section and added more recent studies on the effects of sulfur on vegetable crop yield, stress resistance, and quality.

5. Comment:

This section is generally acceptable, but statistical reporting is insufficient. Since sulfur significantly influences nitrogen assimilation and chlorophyll biosynthesis, more emphasis on how treatments affected these physiological traits would strengthen the section. Lines 178–180: State the statistical significance level used to compare means. Line 186: Specify the significance level (e.g., p < 0.05).

Response:

Thank you for your insightful comment. We have added the significance level for comparing means (p < 0.05) in the manuscript.

6. Comment:

The discussion should be strengthened. Sulfur’s impact on cabbage growth should be explained in terms of its role in amino acid biosynthesis, protein formation, chlorophyll content, and interaction with nitrogen. Since cabbage is a Brassica crop, sulfur’s involvement in glucosinolate synthesis and potential health‑promoting compounds should also be highlighted. Provide mechanistic explanations for the results instead of mainly comparing them with previous studies. Conclude with a clear summary emphasizing the agronomic and nutritional significance of sulfur in cabbage production.

Response:

Thank you for your insightful comment. In response to the comments, we have strengthened the Discussion section. The effects of sulfur on cabbage growth are now explained from the perspectives of amino acid biosynthesis, protein formation, chlorophyll content, and sulfur–nitrogen interactions. We have also discussed the role of sulfur in glucosinolate synthesis and its contribution to potential health‑promoting compounds. The mechanistic aspects of the results are further clarified. As a result, the agronomic and nutritional significance of sulfur in cabbage production has been more prominently highlighted.

7. Comment:

Ensure all references cited in the text appear in the reference list and vice versa. Format references according to PLOS ONE guidelines. Include recent research (within the last 5–7 years) on sulfur nutrition in Brassica crops.

Response:

Thank you for your insightful comment. We have carefully verified all references in the manuscript. All references are now correctly listed in the reference section, and every entry has been revised to comply with the journal’s formatting guidelines. Additionally, we have updated the references to include recent advances, with most citations drawn from studies published in the past five years.

8. Comment:

Round all numerical values to two decimal places. Table 3-1: Revise headings to Chlorophyll a, Chlorophyll b, and Total chlorophyll content. Table 3-2: Revise the title. Use standard abbreviations in headings (e.g., CO₂, Tr, Si, Pn) with units, and explain all abbreviations in the table footnote.

Response:

Thank you for your insightful comment. We have carefully addressed all of the reviewer’s comments on the tables and made the necessary revisions.

9. Comment:

Place figure numbering on the left side of each figure. Figure 3-3: Improve image quality for better clarity.

Response:

Thank you for your insightful comment. We have carefully addressed all of the reviewer’s comments on the figures and made the necessary revisions. The quality of Figure 3.3 is already at its highest. We have tried many approaches but still cannot effectively improve the image quality.

Attachment Submitted filename: PONE ONE.docx

# Peer review: Decision Letter 1

**DOI:** 10.1371/journal.pone.0359848.r003

**Authors:** Trung Quang Nguyen

13 Jul 2026

Dear Dr. Lushan,

Thank you for submitting your manuscript to PLOS ONE. After careful consideration, we feel that it has merit but does not fully meet PLOS ONE’s publication criteria as it currently stands. Therefore, we invite you to submit a revised version of the manuscript that addresses the points raised during the review process.

Please submit your revised manuscript by Aug 27 2026 11:59PM. If you will need more time than this to complete your revisions, please reply to this message or contact the journal office at plosone@plos.org. When you're ready to submit your revision, log on to https://www.editorialmanager.com/pone/ and select the 'Submissions Needing Revision' folder to locate your manuscript file.

- A letter that responds to each point raised by the academic editor and reviewer(s). You should upload this letter as a separate file labeled 'Response to Reviewers'.
- A marked-up copy of your manuscript that highlights changes made to the original version. You should upload this as a separate file labeled 'Revised Manuscript with Track Changes'.
- An unmarked version of your revised paper without tracked changes. You should upload this as a separate file labeled 'Manuscript'.

If you would like to make changes to your financial disclosure, please include your updated statement in your cover letter. Guidelines for resubmitting your figure files are available below the reviewer comments at the end of this letter.

If applicable, we recommend that you deposit your laboratory protocols in protocols.io to enhance the reproducibility of your results. Protocols.io assigns your protocol its own identifier (DOI) so that it can be cited independently in the future. For instructions see: https://journals.plos.org/plosone/s/submission-guidelines#loc-laboratory-protocols. Additionally, PLOS ONE offers an option for publishing peer-reviewed Lab Protocol articles, which describe protocols hosted on protocols.io. Read more information on sharing protocols at https://plos.org/protocols?utm_medium=editorial-email&utm_source=authorletters&utm_campaign=protocols.

As the corresponding author, your ORCID iD is verified in the submission system and will appear in the published article. PLOS supports the use of ORCID, and we encourage all coauthors to register for an ORCID iD and use it as well. Please encourage your coauthors to verify their ORCID iD within the submission system before final acceptance, as unverified ORCID iDs will not appear in the published article. *Only*  the individual author can complete the verification step; PLOS staff *cannot*  verify ORCID iDs on behalf of authors.

We look forward to receiving your revised manuscript.

Kind regards,

Trung Quang Nguyen

Academic Editor

PLOS One

Journal Requirements:

1. If the reviewer comments include a recommendation to cite specific previously published works, please review and evaluate these publications to determine whether they are relevant and should be cited. There is no requirement to cite these works unless the editor has indicated otherwise.

2. Please review your reference list to ensure that it is complete and correct. If you have cited papers that have been retracted, please include the rationale for doing so in the manuscript text, or remove these references and replace them with relevant current references. Any changes to the reference list should be mentioned in the rebuttal letter that accompanies your revised manuscript. If you need to cite a retracted article, indicate the article’s retracted status in the References list and also include a citation and full reference for the retraction notice.

[Note: HTML markup is below. Please do not edit.]

Reviewers' comments:

Reviewer’s Responses to Questions

**Comments to the Author**

Reviewer #2: (No Response)

Reviewer #3: All comments have been addressed

**********

2. Is the manuscript technically sound, and do the data support the conclusions?

Reviewer #2: Yes

Reviewer #3: Yes

**********

3. Has the statistical analysis been performed appropriately and rigorously?

Reviewer #2: I Don't Know

Reviewer #3: Yes

**********

4. Have the authors made all data underlying the findings in their manuscript fully available?

The PLOS Data policy

Reviewer #2: Yes

Reviewer #3: Yes

**********

5. Is the manuscript presented in an intelligible fashion and written in standard English?

Reviewer #2: Yes

Reviewer #3: Yes

**********

Reviewer #2: I observed that different fonts were used in the writing; the publishing team can correct this.

Line 111-114, it will be good to add that MgCl2.7H2O was substituted with MgSO47H20 for samples that had Sulphur

All through 130-154, the kit was mentioned, but not the name of the kit used in the various assays

Line 190 spelling error, please correct to specified

207-208: the PCR step should have the stages: denaturation...

Observed that the statistics in materials and methods state p<0.05, but for the figures, the p<0.5, though the tables have P<0.05

Line 280 spelling error exchange

Line 281 As not AS

Line 406 reads Note: Different lowercase letters............. when the figure had no letters.

Line 419, the double periods should have only one

Line 448, the need for spaces among a few words

Line 528 should begin with a capital letter U

Reviewer #3: This manuscript addresses an important topic and has the potential to contribute to the field. However, several areas require improvement before publication. The authors should provide clearer justification for the study, strengthen the description of the methodology, and ensure that the results and conclusions are fully supported by the data. The discussion should better relate the findings to existing literature and acknowledge study limitations. Additional attention is also needed regarding clarity, organization, and ethical reporting. Overall, the manuscript is promising but requires revision to improve its scientific rigor and presentation.

**********

what does this mean?). If published, this will include your full peer review and any attached files.

If you choose “no”, your identity will remain anonymous but your review may still be made public.

**Do you want your identity to be public for this peer review?**  For information about this choice, including consent withdrawal, please see our Privacy Policy

Reviewer #2: No

Reviewer #3: No

**********

[NOTE: If reviewer comments were submitted as an attachment file, they will be attached to this email and accessible via the submission site. Please log into your account, locate the manuscript record, and check for the action link "View Attachments". If this link does not appear, there are no attachment files.]

To ensure your figures meet our technical requirements, please review our figure guidelines: https://journals.plos.org/plosone/s/figures

You may also use PLOS’s free figure tool, NAAS, to help you prepare publication quality figures: https://journals.plos.org/plosone/s/figures#loc-tools-for-figure-preparation.

NAAS will assess whether your figures meet our technical requirements by comparing each figure against our figure specifications.

Attachment Submitted filename: Comments.docx

# Author comment: Author response to Decision Letter 2

**DOI:** 10.1371/journal.pone.0359848.r004

14 Aug 2026

PONE-D-25-34117 Revision Letter

Journal Name: PLOS ONE

Manuscript Number: PONE-D-25-34117

Manuscript Name: Effects of sulfur on the growth and metabolism of sulfur and nitrogen in cabbage

Initial Date Submitted: Jul 10 2025

Dear Editor:

We greatly appreciate the time and effort you and the reviewers have dedicated to reviewing our manuscript entitled " Effects of sulfur on the growth and metabolism of sulfur and nitrogen in cabbage ". We have carefully considered all the comments and suggestions, and have revised our manuscript accordingly.

Below is a point-by-point response to the reviewers' comments:

1.English language and grammar

Although the language has improved, the manuscript still contains numerous grammatical errors, awkward sentence structures, typographical mistakes, and inconsistent terminology. Examples include "Sulffur," "gas excage parameters," "Photosynthetic chlorophyll fluorescencephotosynthetic parameters," inconsistent spacing around "+S" and "-S," and incorrect article usage throughout the manuscript. A thorough professional English editing is still recommended.

Response:

Thank you for pointing out the remaining language issues. In response to your suggestion, we have engaged a professional scientific editing service to thoroughly revise the entire manuscript. Beyond correcting the grammatical errors, awkward structures, and typographical mistakes you noted (including "Sulffur," "gas excage parameters," and inconsistent terminology), the editor also standardized the format of our treatment labels (+S/-S) and refined the article usage throughout. A copy of the editing certificate has been uploaded as a supplementary file. We are confident that the language quality is now significantly improved.

2.Experimental design

The sulfur treatment is now better described, but the manuscript still lacks sufficient justification for selecting only one sulfur concentration (2 mmol L⁻¹). The authors should explain why this concentration was chosen and discuss the limitation of using only two sulfur levels (deficient vs. sufficient) rather than a dose-response design.

Response:

Thank you for your insightful comment. We appreciate the reviewer’s concern regarding the single sulfur concentration. We would like to clarify that the primary objective of our study was to elucidate the physiological and molecular mechanisms underlying sulfur deficiency stress, rather than to determine the optimal sulfur requirement for cabbage. Therefore, we adopted a binary experimental design (deficient vs. sufficient) to represent two clearly contrasting nutritional statuses, which is a standard approach in stress physiology studies.

The concentration of 2 mmol L⁻¹ corresponds to the standard sulfur supply in the widely used Hoagland nutrient solution formulation. This concentration has been consistently employed in our preliminary cultivation practices and is recognized as a sufficient and non-toxic sulfur level that supports normal growth for a broad range of plant species. Therefore, we selected this concentration as the "sufficient" treatment in our experimental design. We acknowledge that a dose-response design would provide additional valuable information, but our current experimental framework was specifically tailored to compare the "presence vs. absence" of adequate sulfur.

In response to your suggestion, we have revised the Materials and Methods section (The line:111-116) to more clearly justify the selection of 2 mmol L⁻¹ based on prior studies, and we have added a limitation statement in the Discussion acknowledging that our conclusions are confined to these two levels and that extrapolation to other concentrations requires further investigation.

3.Statistical analysis

The statistical analysis remains confusing. The manuscript states that both Tukey’s test and Duncan’s multiple range test were used. The authors should clarify which multiple comparison test was actually applied and use only one method consistently throughout the manuscript.

Response:

We thank the reviewer for identifying this inconsistency. We apologize for the confusion.After re-examining our data and analytical approach, we confirm that Duncan’s multiple range test was the method actually used for all post-hoc comparisons. Although both tests were inadvertently mentioned in the original submission, we have now carefully revised the entire manuscript to consistently use only Duncan’s test.

4.Interpretation of results

Several conclusions remain stronger than the presented evidence. Statements such as sulfur acting as a "central hub" regulating metabolic networks or establishing a "theoretical framework" are overstated because the study mainly presents physiological and biochemical measurements rather than mechanistic or molecular evidence. These conclusions should be moderated.

Response:

We greatly appreciate the reviewer’s thoughtful comments. Accordingly, we have carefully revised both the Introduction and the Conclusion sections to ensure that all descriptions remain strictly focused within the scope of our study. Content that was deemed beyond the scope of this work has been either removed or appropriately rephrased.

5.Gene expression analysis

The qRT-PCR results are briefly presented, but important methodological details remain limited. Information regarding primer validation, amplification efficiency, reference gene stability, and biological replication should be described more clearly.

Response:

We sincerely thank you for your careful review and valuable comments on our manuscript. We have thoroughly considered your suggestions and have revised the manuscript accordingly. Below we provide our point-by-point responses to your concerns.

We fully agree with the reviewer’s assessment that methodological details are critical for the reproducibility of qRT-PCR results. Accordingly, we have significantly expanded the description of our qRT-PCR methodology in the Materials and Methods section.

Specifically, we have now added the following details:

Primer design and validation: All primers were designed using Primer Premier 5.0 based on the cDNA sequences deposited in GenBank.

Amplification efficiency: All primers used in this study exhibited amplification efficiencies ranging from 92% to 105% (R² > 0.99).

Replication: All qRT-PCR reactions were performed in triplicate technical repeats, and three independent biological replicates were conducted for each treatment.

Quantification method: The relative expression levels of target genes were calculated using the 2⁻ΔΔCt method after normalization to the selected reference gene.

We believe these additions substantially improve the methodological transparency and reproducibility of our study. We are grateful for the reviewer’s constructive suggestion, which has helped us strengthen our manuscript.

6.Discussion

The discussion has improved considerably, but some sections remain descriptive. More emphasis should be placed on explaining the physiological mechanisms underlying sulfur-mediated regulation of nitrogen metabolism and antioxidant responses rather than simply comparing findings with previous reports.

Response:

We sincerely thank the reviewer for this insightful and constructive comment. We fully agree that a deeper mechanistic explanation is far more valuable than simple data comparison. Accordingly, we have substantially revised the Discussion section with the following improvements:

In the nitrogen metabolism subsection, we have elucidated the mechanistic influence of sulfur on nitrogen-metabolizing enzymes. Sulfur operates through a sophisticated regulatory network involving three interconnected mechanisms: feedback regulation by sulfur-containing amino acids (cysteine), signal transduction by the metabolic intermediate O-acetylserine (OAS), and oxidative-reductive modification of enzyme proteins. When sulfur supply is adequate, cysteine synthesis increases, and its accumulation exerts feedback inhibition to downregulate the activities and gene expression of key nitrogen-metabolizing enzymes such as nitrate reductase (NR) and glutamine synthetase (GS), thereby preventing ammonium toxicity caused by excessive nitrogen assimilation. Conversely, under sulfur deficiency, cysteine synthesis is blocked, leading to accumulation of its precursor OAS, which acts as a potent signaling molecule to activate sulfur transport gene expression while simultaneously relieving the inhibition on NR and even promoting its gene transcription, driving the plant to absorb more nitrate in a "search" for sulfur—thus coordinating nitrogen-sulfur balance. In addition, the redox status of thiol groups (-SH) on enzyme molecules such as NR is subject to regulation by cellular reducing power, directly and rapidly modulating the enzymatic activity switch, and the molybdenum cofactor, for whose synthesis sulfur is essential, is also a necessary component for NR activity.

In the antioxidant response subsection, we have elucidated that sulfur, in the form of thiol groups, acts as a redox "sensor" on the Keap1 protein. When the organism is subjected to oxidative stress, these thiol groups are modified, prompting Keap1 to release the transcription factor Nrf2. Once activated, Nrf2 translocates to the nucleus and initiates the expression of downstream antioxidant enzymes (such as SOD, CAT, GPx, etc.). These enzymes efficiently scavenge free radicals to protect cell membranes from lipid peroxidation attack, and the ultimate effect is directly reflected as a significant reduction in the production of malondialdehyde (MDA), a terminal product of lipid peroxidation. Therefore, within this complete signaling chain, sulfur serves as the initial trigger, Nrf2 as the central commander, antioxidant enzymes as the front-line executors, and MDA as a marker of ultimate damage extent—together forming a closed-loop antioxidant defense mechanism of "sulfur modification → pathway activation → enzymatic scavenging → damage reduction."

All revisions have been using track changes for your easy reference. We hope these modifications adequately address your concern.

7.Minor comments

Carefully check all statistical significance statements. Several figure legends still report "p < 0.5" instead of "p < 0.05."

Verify consistency in terminology (photosynthetic parameters, chlorophyll fluorescence parameters, sulfur metabolism, nitrogen metabolism).

Ensure all abbreviations are defined upon first appearance.

Several tables require formatting improvement, including consistent units and spacing.

Figure legends should be edited for clarity and grammatical correctness.

Some references cited in the discussion should be checked to ensure they directly support the corresponding statements.

The manuscript would benefit from careful proofreading to remove remaining formatting artifacts introduced during revision.

Response:

We sincerely thank the reviewer for this thorough and meticulous review. These technical and editorial points are extremely valuable for improving the overall quality and clarity of our manuscript. We have carefully addressed each issue as detailed below:

1. Statistical significance statements. We have meticulously checked all significance indicators throughout the manuscript. All instances of "p < 0.5" have been corrected to the proper threshold of "p < 0.05" in both the text and all figure legends. We have also verified that all other statistical notations (e.g., p < 0.01, p < 0.001) are correctly reported.

2. Terminology consistency. We have systematically reviewed the entire manuscript to ensure consistent usage of the following key terms: photosynthetic parameters (e.g., Pn, Gs, Ci, Tr), chlorophyll fluorescence parameters (e.g., Fv/Fm, ΦPSII, qP, NPQ), sulfur metabolism (e.g., sulfate uptake, cysteine biosynthesis, glutathione pool), and nitrogen metabolism (e.g., nitrate reductase, glutamine synthetase, nitrogen assimilation). All terms are now used uniformly throughout the text, tables, and figures.

3. Abbreviation definitions. We have verified that every abbreviation is defined upon its first appearance in the abstract, main text, and each figure legend. A comprehensive list of abbreviations has also been added (or updated) for easy reference.

4. Table formatting. All tables have been reformatted to improve readability. We have ensured consistent units are provided for all parameters and that appropriate spacing has been applied between columns and rows. Units are now uniformly placed in the column headers rather than repeated in each cell.

5. Figure legends. We have carefully edited all figure legends for clarity and grammatical correctness. Redundant or ambiguous descriptions have been revised to ensure each legend is self-explanatory and accurately reflects the corresponding figure content.

6. Reference verification. We have re‑examined all references cited in the Discussion section to confirm that each one directly supports the corresponding statement. Where necessary, we have replaced or supplemented references with more relevant citations.

7. Proofreading. The entire manuscript has undergone careful proofreading to remove formatting artifacts and typographical errors introduced during the revision process. We have also checked line spacing, font consistency, and reference formatting.

All revisions have been using track changes for your easy reference. We hope these improvements meet your expectations.

Attachment Submitted filename: Response to reviewers(new1).docx

# Peer review: Decision Letter 2

**DOI:** 10.1371/journal.pone.0359848.r005

**Authors:** Trung Quang Nguyen

20 Sep 2026

<p>Effects of sulfur on the growth and metabolism of sulfur and nitrogen in cabbage

PONE-D-25-34117R2

Dear Dr. Li Lushan,

We’re pleased to inform you that your manuscript has been judged scientifically suitable for publication and will be formally accepted for publication once it meets all outstanding technical requirements.

Within one week, you’ll receive an e-mail detailing the required amendments. When these have been addressed, you’ll receive a formal acceptance letter and your manuscript will be scheduled for publication.

An invoice will be generated when your article is formally accepted. Please note, if your institution has a publishing partnership with PLOS and your article meets the relevant criteria, all or part of your publication costs will be covered. Please make sure your user information is up-to-date by logging into Editorial Manager at Editorial Manager® and clicking the ‘Update My Information' link at the top of the page. For questions related to billing, please contact billing support.

If your institution or institutions have a press office, please notify them about your upcoming paper to help maximize its impact. If they’ll be preparing press materials, please inform our press team as soon as possible -- no later than 48 hours after receiving the formal acceptance. Your manuscript will remain under strict press embargo until 2 pm Eastern Time on the date of publication. For more information, please contact onepress@plos.org.

Kind regards,

Trung Quang Nguyen

Academic Editor

PLOS One

Additional Editor Comments (optional):

Reviewers' comments:

Reviewer’s Responses to Questions

**Comments to the Author**

Reviewer #2: All comments have been addressed

**********

2. Is the manuscript technically sound, and do the data support the conclusions?

Reviewer #2: Yes

**********

3. Has the statistical analysis been performed appropriately and rigorously?

Reviewer #2: Yes

**********

4. Have the authors made all data underlying the findings in their manuscript fully available?

The PLOS Data policy

Reviewer #2: No

**********

5. Is the manuscript presented in an intelligible fashion and written in standard English?

Reviewer #2: Yes

**********

Reviewer #2: (No Response)

**********

what does this mean?). If published, this will include your full peer review and any attached files.

If you choose “no”, your identity will remain anonymous but your review may still be made public.

**Do you want your identity to be public for this peer review?**  For information about this choice, including consent withdrawal, please see our Privacy Policy

Reviewer #2: No

**********

# Editor report: Acceptance letter

**DOI:** 10.1371/journal.pone.0359848.r006

**Authors:** Trung Quang Nguyen

PONE-D-25-34117R2

PLOS One

Dear Dr. Li,

I'm pleased to inform you that your manuscript has been deemed suitable for publication in PLOS One. Congratulations! Your manuscript is now being handed over to our production team.

At this stage, our production department will prepare your paper for publication. This includes ensuring the following:

* All references, tables, and figures are properly cited

* All relevant supporting information is included in the manuscript submission,

* There are no issues that prevent the paper from being properly typeset

You will receive further instructions from the production team, including instructions on how to review your proof when it is ready. Please keep in mind that we are working through a large volume of accepted articles, so please give us a few days to review your paper and let you know the next and final steps.

Lastly, if your institution or institutions have a press office, please let them know about your upcoming paper now to help maximize its impact. If they'll be preparing press materials, please inform our press team within the next 48 hours. Your manuscript will remain under strict press embargo until 2 pm Eastern Time on the date of publication. For more information, please contact onepress@plos.org.

You will receive an invoice from PLOS for your publication fee after your manuscript has reached the completed accept phase. If you receive an email requesting payment before acceptance or for any other service, this may be a phishing scheme. Learn how to identify phishing emails and protect your accounts at https://explore.plos.org/phishing.

If we can help with anything else, please email us at customercare@plos.org.

Thank you for submitting your work to PLOS One and supporting open access.

Kind regards,

PLOS One Editorial Office Staff

on behalf of

Dr. Trung Quang Nguyen

Academic Editor

PLOS One
